# Distinct early cellular kinetics in participants protected against colonization upon *Bordetella pertussis* challenge

**DOI:** 10.1172/JCI163121

**Published:** 2023-03-01

**Authors:** Annieck M. Diks, Hans de Graaf, Cristina Teodosio, Rick J. Groenland, Bas de Mooij, Muktar Ibrahim, Alison R. Hill, Robert C. Read, Jacques J.M. van Dongen, Magdalena A. Berkowska, on behalf of the IMI-2 PERISCOPE Consortium

**Affiliations:** 1 Department of Immunology, Leiden University Medical Center, Leiden, Netherlands.; 2 Faculty of Medicine and; 3 NIHR Southampton Biomedical Research Centre, University of Southampton, University Hospital Southampton NHS Foundation Trust, Southampton, United Kingdom.; 4 Centro de Investigación del Cáncer-Instituto de Biología Molecular y Celular del Cáncer (CIC-IBMCC, USAL-CSIC-FICUS) and Department of Medicine, University of Salamanca, Salamanca, Spain.; 5 The IMI-2 PERISCOPE Consortium is detailed in Supplemental Acknowledgments.

**Keywords:** Immunology, Infectious disease, Adaptive immunity, Bacterial infections, Innate immunity

## Abstract

**BACKGROUND:**

To date, only limited data are available on the mechanisms of protection against colonization with *Bordetella pertussis* in humans.

**METHODS:**

In this study, the cellular responses to *B*. *pertussis* challenge were monitored longitudinally using high-dimensional EuroFlow-based flow cytometry, allowing quantitative detection of more than 250 different immune cell subsets in the blood of 15 healthy donors.

**RESULTS:**

Participants who were protected against colonization showed different early cellular responses compared with colonized participants. Especially prominent for colonization-protected participants were the early expansion of CD36^^–^^ nonclassical monocytes on day 1 (D1), natural killer cells (D3), follicular T helper cells (D1–D3), and plasma cells (D3). Plasma cell expansion on D3 correlated negatively with the CFU load on D7 and D9 after challenge. Increased plasma cell maturation on D11–D14 was found in participants with seroconversion.

**CONCLUSION:**

These early cellular immune responses following experimental infection can now be further characterized and potentially linked to an efficient mucosal immune response, preventing colonization. Ultimately, their presence may be used to evaluate whether new *B*. *pertussis* vaccine candidates are protective against *B*. *pertussis* colonization, e.g., by bacterial challenge after vaccination.

**TRIAL REGISTRATION:**

ClinicalTrials.gov NCT03751514.

**FUNDING:**

Innovative Medicines Initiative 2 Joint Undertaking and the EuroFlow Consortium.

## Introduction

The introduction of vaccines has significantly reduced the amount of infectious disease–related deaths (1). However, vaccine-induced protection is often based on empirical results, and the mechanisms of protection and underlying immunological processes are not completely understood. Generally, the determination of antigen-specific immunoglobulin (Ag-specific Ig) levels in the serum is used as a readout for vaccine efficacy or protective immunity. For vaccines/diseases for which no true correlate of protection is defined, as is the case for pertussis, increased insight into the immunological processes associated with infection may be an important step forward. Pertussis is a disease of the respiratory tract, caused by the bacterium *Bordetella pertussis*. Infection with *B*. *pertussis* occurs via respiratory droplets. Although the whole-cell pertussis (wP) vaccine and the acellular pertussis (aP) vaccine are known to protect against disease, the incidence of pertussis has been rising in recent decades (2). Several animal models have been established to study pertussis infection and vaccination. These models resulted in valuable insights into the different immune responses induces by aP and wP vaccination and new vaccine candidates (including utilizing different routes of administration), and infection (3–5). In baboons, aP vaccination induced a T helper 1 (Th1)/Th2 response, protecting animals from severe disease, but did not prevent colonization and transmission to naive animals (3). In contrast, natural infection with *B*. *pertussis*, and to a lesser extent wP vaccination, induced a Th1/Th17 memory response. Moreover, wP-vaccinated animals were protected from severe disease, and although they were not protected against colonization, they cleared the infection significantly more quickly than aP-vaccinated and naive animals (3). Lastly, previously infected animals were protected from colonization and disease (3). The question remains to what extent animal models resemble the course of *B*. *pertussis* infection and the accompanying immune response in humans. This topic can only be addressed in human vaccination and challenge studies.

Data on humans in the pre–pertussis vaccine era indicated that although natural infection does not result in complete lifelong protection, reinfection is much milder than primary infection (6). Moreover, protection after natural infection was estimated to last longer compared with aP or wP vaccine–induced protection (6). This implies that despite high vaccination coverage and the induction of Ag-specific Igs (especially against pertussis toxin, PT), the carriage and transmission of pertussis persists, which may explain the reported resurgence of pertussis in recent decades (7–9). Improved protection against transmission is an important aspect for the development of future vaccines/vaccination strategies against pertussis.

Currently, modifications of existing pertussis vaccines as well as new vaccine candidates are being investigated (4). Yet, limited data are available on the mechanisms and immune signatures associated with protection against colonization. Moreover, it is difficult to extrapolate data from intramuscular vaccination to other routes of administration, such as intranasal delivery, which more closely resembles natural *B*. *pertussis* encounters. For pertussis, the live-attenuated BPZE1 vaccine, which is delivered intranasally, is currently one of the most advanced vaccine candidates in clinical development (4, 10).

In 2017, a human challenge model for *B*. *pertussis* (controlled bacterial infection to induce colonization) was established at the National Health Institute for Health Research Clinical Research Facility Southampton, United Kingdom (UK), by the University of Southampton as part of the IMI-2 PERISCOPE Consortium (11, 12). In this model, asymptomatic colonization of participants can be established in a safe manner. A major advantage of using this human challenge model is that one can define more clearly whether a participant is protected against colonization by culturing bacteria from throat and nasal samples. Presence and density of these bacteria can be further correlated with the levels of antibodies in serum. Moreover, in contrast to sampling from pertussis patients, the exact moment of infection is known, and the synchronized sampling in multiple participants enables us to find shared patterns arising after bacterial challenge. The analysis of the cellular immune response following bacterial challenge may yield new insights into the cellular kinetics important for early removal of bacteria from the mucosal layers and protection against colonization. Comparison of similarities and differences between the immune responses launched after vaccination or bacterial challenge may help to identify why current pertussis vaccines do not fully protect against infection and what should be changed to induce protection against carriage. Identification of immune signatures associated with protection against colonization may be of importance in the evaluation of vaccines that aim at reduction/prevention of carriage and transmission.

In this exploratory study, we monitored the cellular kinetics in humans after intranasal challenge with *B*. *pertussis*. We wanted to evaluate whether the immune system is systemically triggered by pathogen encounter, and whether protection from colonization can be predicted by the magnitude of this response, involvement of particular cell subsets, or an overall immune signature. All participants were primed with wP and had no known recent contact with *B*. *pertussis* (vaccination against *B*. *pertussis* had to be at least 5 years prior to the study). We monitored the immune responses in these participants and identified cellular changes that were unique for participants protected from colonization.

## Results

### Ten of 15 participants colonized after challenge with B1917 strain.

**Figure 1:**
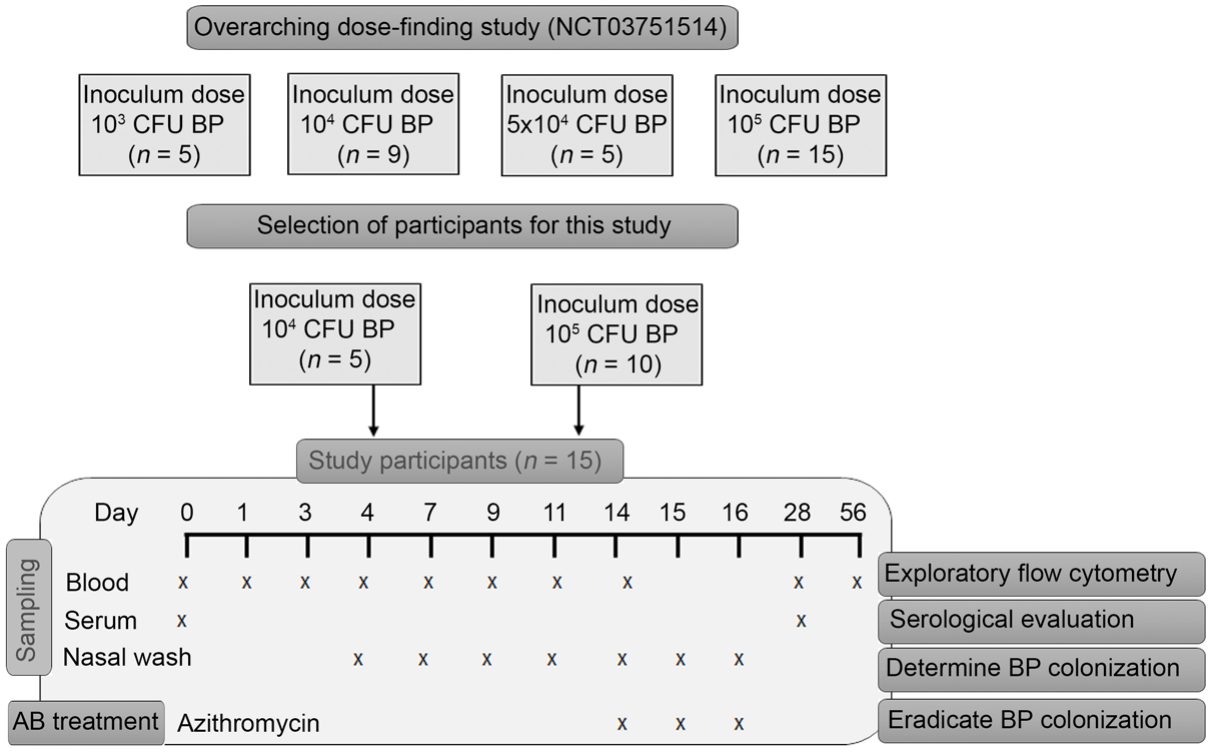
Study and cohort description and clinical readout. Overview of study set up, intervention, and sampling time points used. Day 0 is the day on which participants were challenged intranasally. AB, antibiotics; BP, *Bordetella pertussis*.

Samples from 15 participants were collected between November 2017 and September 2018. Five participants were challenged intranasally with 1 × 10^4^ colony forming units (CFU) *B*. *pertussis* strain B1917 and 10 participants were challenged intranasally with 1 × 10^5^ CFU (Figure 1). As reported previously, participants challenged with 1 × 10^5^ CFU more frequently reported mild symptoms of cough and rhinorrhea and nasal congestion than participants challenged with 1 × 10^4^ CFU (12). None of the participants developed pertussis disease or required rescue medication. No serious adverse events were reported. To ensure that the different inoculum dose does not influence the immune cell kinetics, we first compared the kinetics of the 1 × 10^4^ and 1 × 10^5^ cohort. No statistically significant differences were observed between the cohorts, leading to the decision to merge these cohorts. The most prominent, but not statistically significant difference between the cohorts was in the number of plasma cells on day 11 (D11) and D14. This trend is indicated in the corresponding result section regarding B cell kinetics. One participant withdrew from the study after D14, and one participant withdrew after D28, both for reasons not related to the study. Samples collected until the moment of withdrawal were used for analysis.

To determine the colonization status after challenge, nasal washes were performed from D4 onwards as described previously (Figure 1) (12). Challenge with 1 × 10^4^ CFU and 1 × 10^5^ CFU resulted in *B*. *pertussis*–positive culture in 2 of 5 and 8 of 10 participants, respectively (Table 1). Of the 10 colonized participants, 4 showed low-density colonization (defined as <1000 CFU/mL at any time point during the study), and 6 showed high-density colonization (>1000 CFU/mL at any time point during the study) (Table 1). In further analyses, we divided the participants based on colonization and seroconversion status and searched for unique immune signatures associated with these readouts.

**Figure 2:**
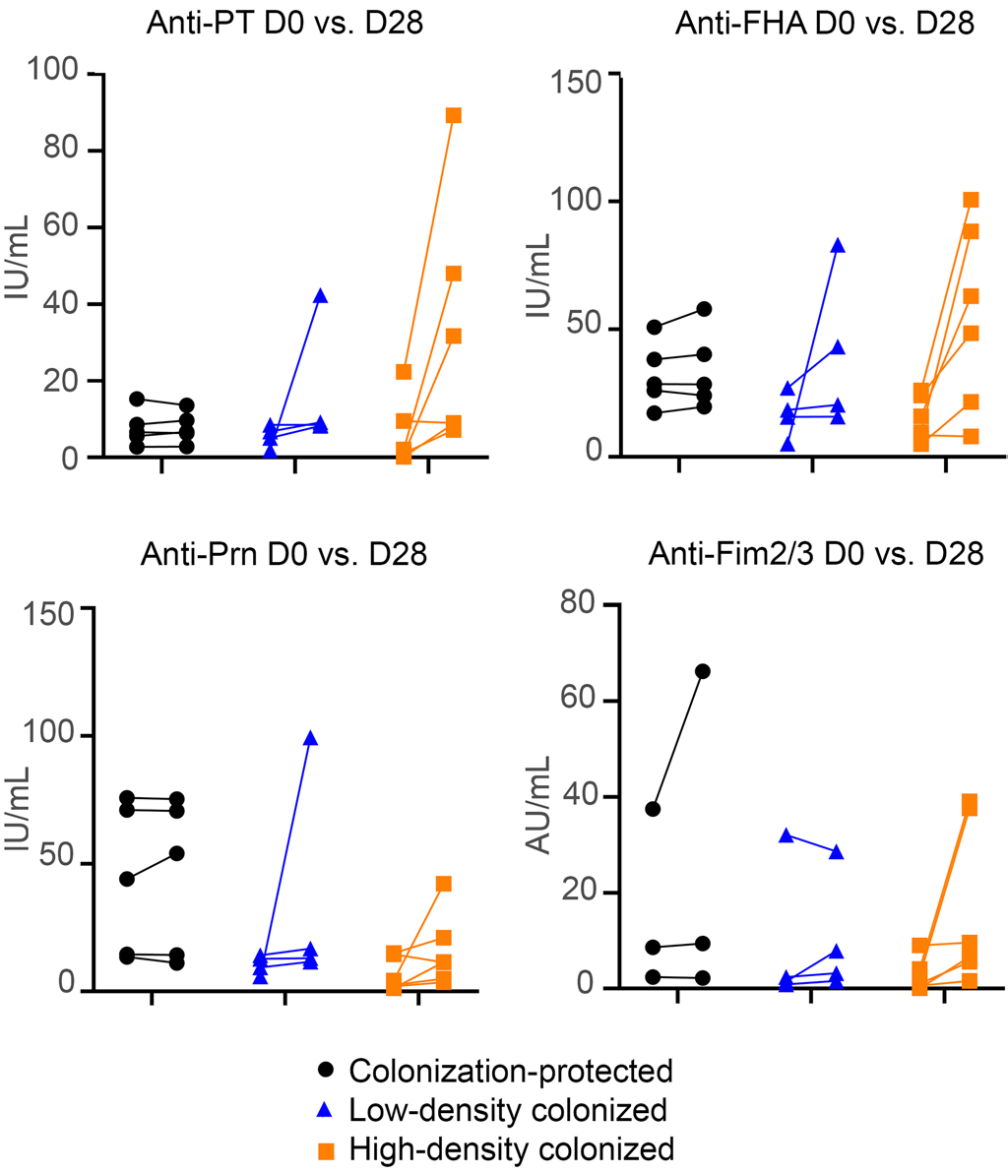
Ag-specific serum IgG levels at baseline (D0) and 28 days after challenge (D28), as evaluated by multiplex immunoassay. Data are arranged according to degree of colonization. IU, international units; AU, arbitrary units; PT, pertussis toxin; FHA, filamentous hemagglutinin; Prn, pertactin, Fim2/3, fimbriae 2 and 3. *n* = 15 (5 colonization-protected, 4 low-density colonized, 6 high-density colonized).

Ag-specific serum IgG levels (against PT, filamentous hemagglutinin [FHA], pertactin [Prn], and fimbriae 2 and 3 [Fim2/3]; expressed as IU/mL) were determined at baseline and on D28 (Figure 2). Comparison of baseline Ag-specific serum IgG levels of the 15 participants evaluated in this study versus all participants included in the overarching bacterial challenge study showed no differences (data not shown). Six participants showed signs of seroconversion (1 low-density- and 5 high-density-colonized participants), while participants protected against colonization showed no sign of seroconversion (Table 1). On top of increased anti-PT IgG serum levels, colonized participants also showed generally higher increases in other Ag-specific IgGs than participants protected against colonization (Figure 2). As only a greater than 2-fold increase in anti-PT serum IgG was used as cutoff for seroconversion, we also investigated whether any participant had a greater than 2-fold increase for another antigen. This was not observed. Lastly, the absolute increase in Ag-specific serum IgG was most prominent in high-density-colonized participants, especially for anti-PT and anti-FHA IgG (Figure 2).

### Fluctuations in circulating innate immune cells after bacterial challenge.

During natural encounter or challenge, the bacterium itself triggers innate immunity at the site of infection (mucosal surfaces); moreover, PT is known to induce systemic effects (13). Thus, a fast (and local) innate response may be important to control *B*. *pertussis* directly upon encounter. As recruitment of innate cells to and from tissues may be detected in the blood early after challenge, we evaluated the cellular changes of innate immune cell subsets after bacterial challenge.

**Figure 3:**
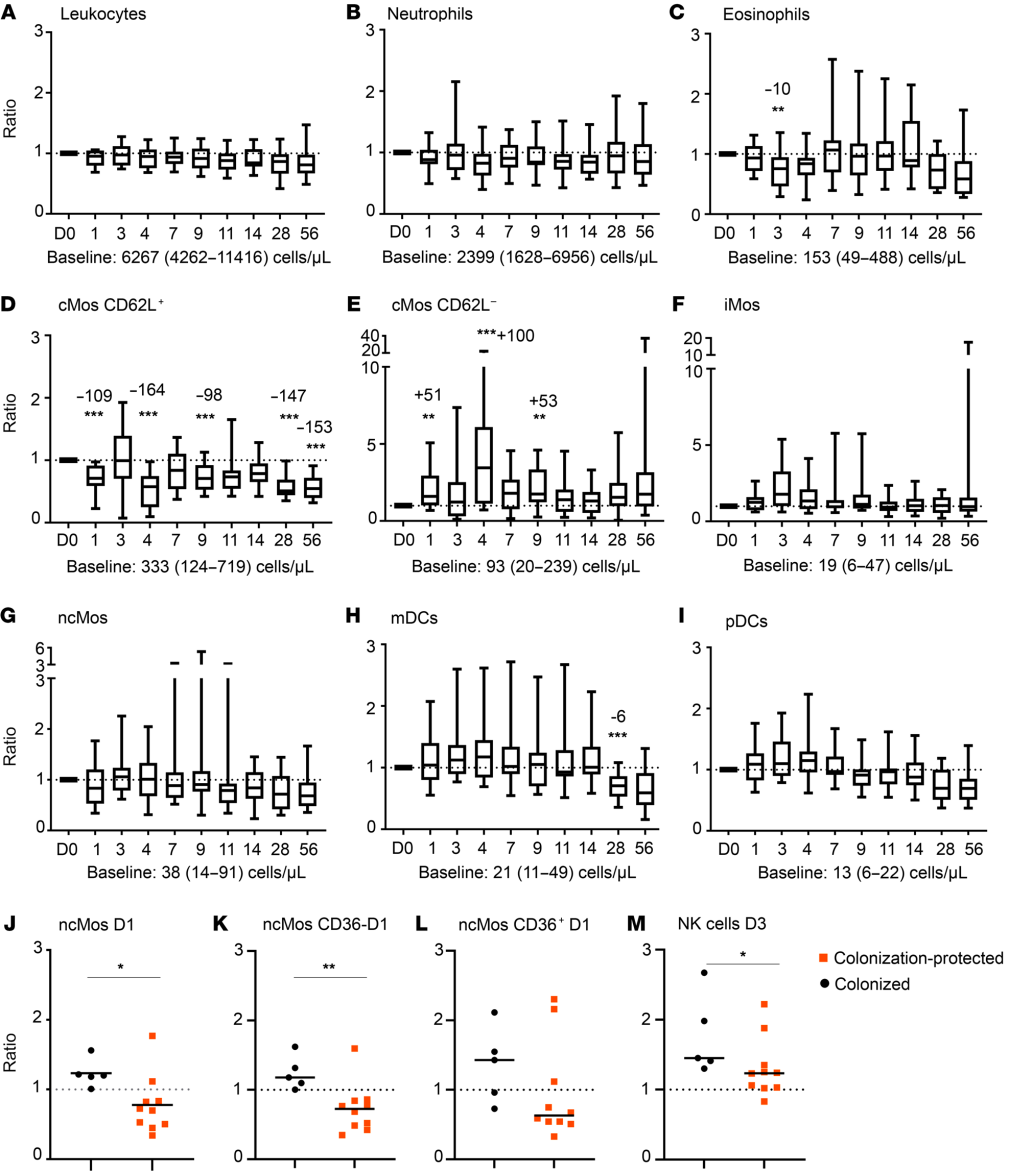
Fluctuations in cell numbers in the innate immune cell compartment after bacterial challenge. Box-and-whisker plots (median, Q1, Q3, min-max) showing the kinetics of major innate immune cell subsets (**A**–**I**) expressed as ratio compared with baseline. *n* = 15. Dashed line indicates a ratio of 1.0 (baseline value). Baseline cell counts for the entire cohort, median (min-max) in cells/μL are indicated below each graph and merely added as an indication of frequency of the cell population. Longitudinal changes were evaluated using Wilcoxon’s matched-pairs signed-rank test and ratio versus baseline. Correction for multiple testing was performed using Bonferroni’s post hoc test. In case of significant differences in ratio compared with baseline, the median increase or decrease in cells/μL is indicated on top of the bar. (**J**–**M**) Increase or decrease in cell numbers expressed as ratio compared to baseline in colonized and colonization-protected participants. *n* = 15. Statistical test performed: Mann-Whitney *U* test on ratio of baseline. *P < 0.05; **P < 0.01; ***P < 0.001. D, days after challenge; cMo, classical monocyte; iMo, intermediate monocyte; ncMo, nonclassical monocyte; mDC, myeloid dendritic cell; pDC, plasmacytoid dendritic cell. Note: Although panel **E** shows that the ratio versus baseline was increased, the median cell count on D28 versus median cell count at baseline was decreased (–7 cells/μL).

Total numbers of circulating leukocytes and neutrophils (expressed as ratio vs. baseline) did not change after bacterial challenge (Figure 3, A and B). Circulating eosinophils decreased on D3 after challenge (Figure 3C). The most prominent changes were observed in circulating monocyte populations. Monocytes mature from classical monocytes (cMos) via intermediate (iMos) to nonclassical monocytes (ncMos) and can be further subdivided into different functional subsets or activation stages (14, 15). Fluctuations in total cMos were limited, with only a minor reduction in circulating cMos on D11 and D28 (data not shown). However, within cMo subsets, decreased numbers (ratio vs. baseline) were observed for CD62L^+^ cMos on D1, D4, D9, D11, D14, D28, and D56 after challenge, whereas increased numbers (ratio vs. baseline) were observed for CD62L^–^ cMos on D1, D4, and D9 after challenge (Figure 3, D and E). CD62L, also known as L-selectin, is shed upon activation and CD62L^–^ cMos are considered activated and possibly more mature (16). No consistent fluctuations were observed for iMos and ncMos (Figure 3, F and G). Within the dendritic cell (DC) compartment, no early consistent changes were observed, although myeloid DCs (mDCs) showed a decrease in cells on D28 after challenge (Figure 3, H and I).

To investigate whether the kinetics of innate immune cells are associated with colonization, we compared the numbers of circulating innate immune cells in participants who were colonized and participants who were protected against colonization. Similar to the collective kinetics, we assessed the longitudinal changes per cell population per colonization status. In order to avoid presenting an excess of data, only the time points where cell populations significantly differed from each other are shown in this paper. No consistent differences between colonized and colonization-protected participants were observed for leukocytes, neutrophils, eosinophils, iMos, cMos, and DCs. An increase in circulating ncMos (ratio vs. baseline), especially CD36^–^ ncMos, was found on D1 after challenge in colonization-protected participants (Figure 3, J–L), while these were decreased in colonized participants. Finally, on D3 after challenge, colonization-protected participants had higher natural killer (NK) cell expansion (ratio vs. baseline) than colonized participants (Figure 3M).

Next, we divided the colonized participants based on colonization density (low- or high-density colonization). Here, we found that the decrease in (especially CD36^–^) ncMos was most prominent for low-density-colonized participants (Supplemental Figure 1, A–C; supplemental material available online with this article; https://doi.org/10.1172/JCI163121DS1). cMo maturation (represented by shedding of CD62L) did not differ between low- and high-density-colonized participants (data not shown). However, we did observe a decrease in a circulating mDC subset in low-density-colonized participants (CD1c^+^CD14^dim^ mDCs; Supplemental Figure 1D). Lastly, NK cells in high-density-colonized participants did not expand, whereas the NK cell expansion in low-density-colonized participants to some extent resembled that of colonization-protected participants (Supplemental Figure 1E).

To summarize, we observed fluctuations in innate cell numbers in the early days after bacterial challenge. Decreased cell numbers may indicate migration of cells into the tissue, and the shedding of CD62L may indicate a shift toward a more activated phenotype in the cMo compartment. Cellular kinetics differed between colonization-protected and colonized participants, and between high- and low-density-colonized participants. The colonization density did not reflect the magnitude of cellular changes, as these were usually more prominent in low-density-colonized participants.

### Limited fluctuations in T cell populations after challenge.

Activation of T cells is required for cellular immunity and the activation of humoral immunity by providing T cell help to B cells in the germinal center reaction (17). As there are different T cell responses generated after vaccination with aP or wP and (natural) infection (18, 19), we set out to determine changes in circulating T cells after experimental exposure to *B*. *pertussis*.

**Figure 4:**
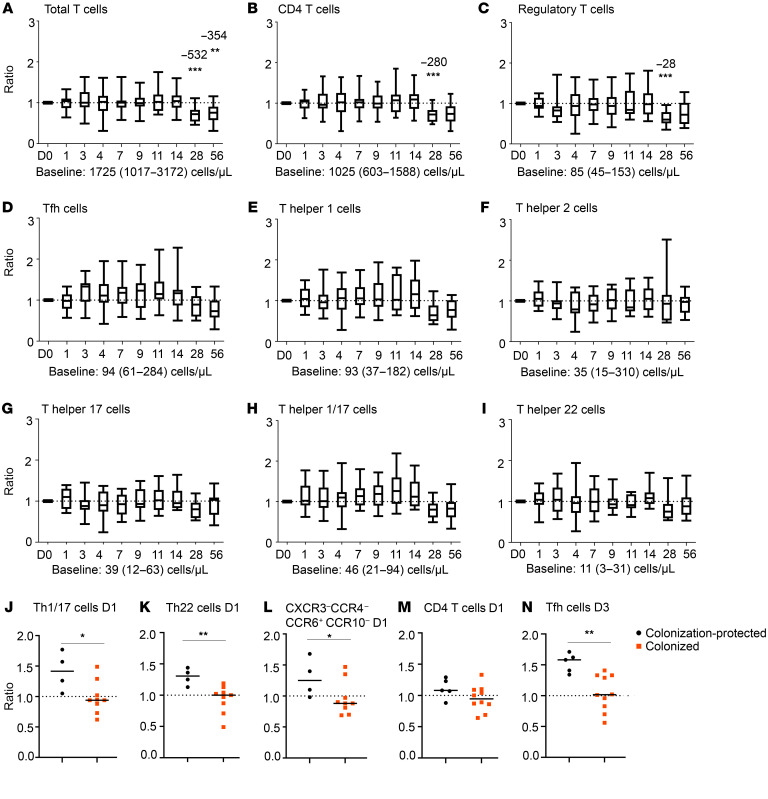
Fluctuations in cell numbers in the T cell compartment after bacterial challenge. Box-and-whisker plots (median, Q1, Q3, min-max) showing the kinetics of major innate immune cell subsets (**A**–**I**) expressed as ratio compared to baseline. *n* = 15. Dashed line indicates a ratio of 1.0 (baseline value). Baseline cell counts for the entire cohort, median (min-max) in cells/μL are indicated below each graph and merely added as an indication of frequency of the cell population. Longitudinal changes were evaluated using Wilcoxon’s matched-pairs signed-rank test and ratio versus baseline. Correction for multiple testing was performed using Bonferroni’s post hoc test. In case of significant differences in ratio compared with baseline, the median increase or decrease in cells/μL is indicated on top of the bar. (**J**–**N**) Increase or decrease in cell numbers expressed as ratio compared to baseline in colonized and colonization-protected participants. *n* = 13 (**J**–**L**) or *n* = 15 (**M** and **N**). Statistical test performed: Mann-Whitney *U* test on ratio of baseline. *P < 0.05; **P < 0.01; ***P < 0.001. D, days after challenge; Tfh, follicular T helper cells; Th, T helper cells; CXCR3^–^CCR4^–^CCR6^+^CCR10^–^ = recently defined Th subset phenotype (20).

Within circulating total T cells, CD4^+^ T cells, CD4^+^ Th, and regulatory T cells, no changes occurred until D28 and D56, when a significant decrease compared with baseline was observed on D28 for CD4^+^ T cells and regulatory T cells, and on D28 and D56 for total T cells (Figure 4, A–C). In follicular T helper (Tfh) cells, no changes were observed after challenge (Figure 4D). Additionally, no consistent changes were observed in CD4^+^ Th cell fluctuations (Figure 4, E–I) or Th cell maturation/activation (cell counts presented in Supplemental Table 1).

When grouping the participants based on colonization status, 3 different CD4^+^ Th subsets increased significantly in colonization-protected participants (ratio vs. baseline) compared with colonized participants on D1 after challenge (Figure 4, J–L). A small expansion in Th1/Th17 cells (CXCR3^+^CCR4^–^CCR6^+^CCR10^–^; recently described by Botafogo et al., ref. 20) was observed on D1 after challenge. Additionally, we found an expansion of Th22 cells and of the recently defined CXCR3^–^CCR4^–^CCR6^+^CCR10^–^ Th subset on D1 in colonization-protected participants. The difference between colonized and colonization-protected participants was not fully explained by total CD4^+^ T cell kinetics (Figure 4M). An increase in total Tfh cells on D3 was primarily found in participants that were protected against colonization (Figure 4N). Due to technical and biological reasons (missing antibody in a surface stain master mix and/or CD45RA polymorphism; ref. 21), at this time point Tfh subsets could only be defined in 9 of 15 participants. Nevertheless, we observed a trend toward increased naive Tfhs, Th1-like, Th1/Th17-like, and Th17-like Tfhs on D3 after challenge in participants that were protected against colonization. This increase was significant for Th2-like Tfh cells (Supplemental Figure 2). When looking at the impact of colonization density, comparison between colonization-protected and low-density-colonized participants resulted in significant differences for Th cell subsets on D1 (Supplemental Figure 3, A–D). Moreover, Tfh expansion on D3 was higher in colonization-protected participants compared with both low- and high-density-colonized participants (Supplemental Figure 3E). No consistent changes were observed in maturation/activation of Th subsets.

Thus, although we observed consistent changes in the Th cell compartment on D1 after bacterial challenge, the most prominent change was the expansion of circulating Tfh cells on D3 after challenge. This expansion was only observed in participants protected against colonization and not polarized to any subset.

### Increased plasma cell numbers on D3 and D11–D14 after challenge.

T cell help, given by Tfh cells in the secondary lymphoid organs, is required to activate B cells after Ag encounter (22). Activation of B cells leads to the formation of memory B cells and plasma cells, and consequently to the production of (protective) antibodies. After (booster) vaccination with aP, skewing toward IgG1 plasma cells has been reported, whereas natural encounter is thought to induce IgA memory, as shown by the positive correlation between age and IgA responses against *B*. *pertussis* (23–26).

**Figure 5:**
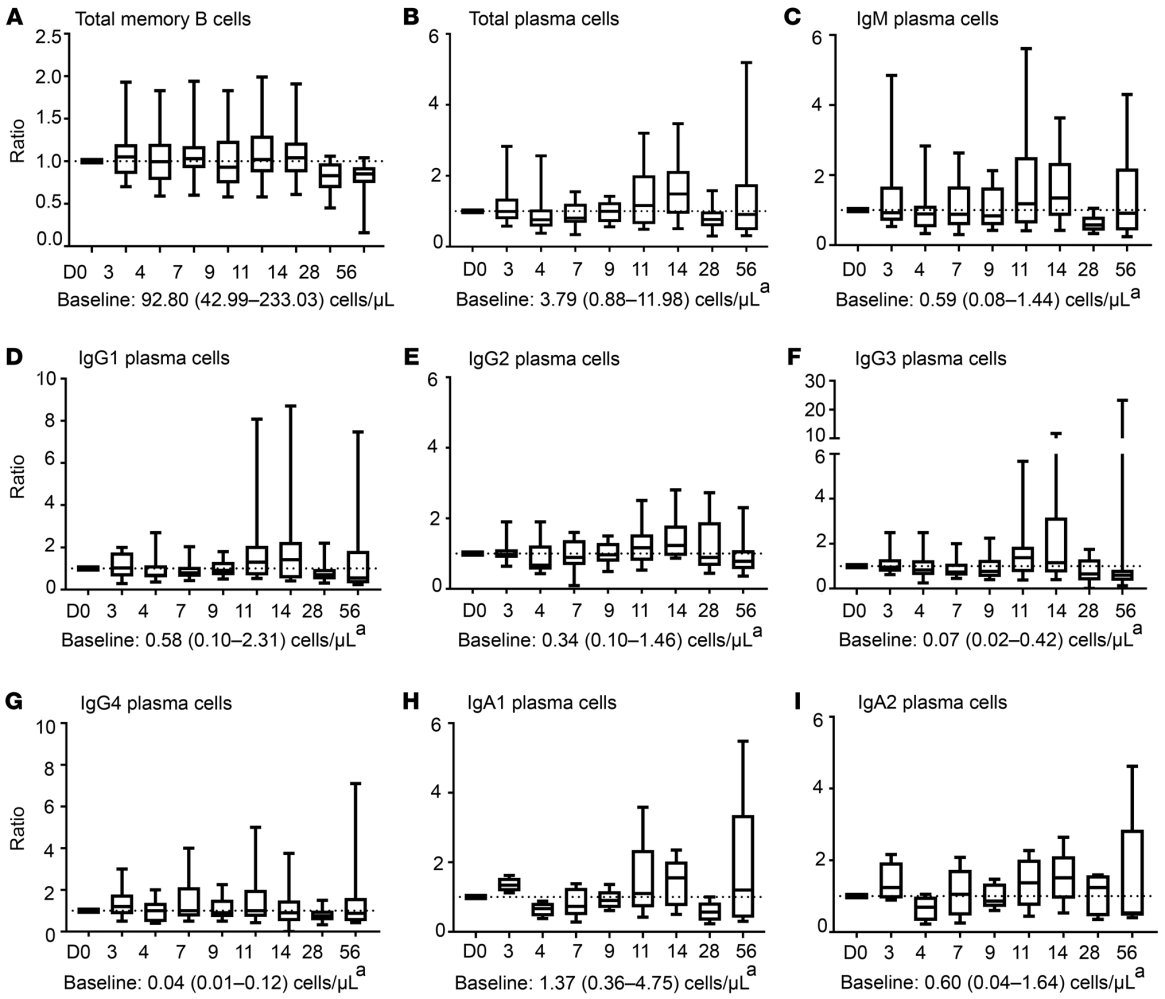
Kinetics in the plasma cell compartment upon bacterial challenge. (**A**) Box-and-whisker plot (median, Q1, Q3, min-max) showing the expansion of total memory B cells after challenge. *n* = 15. (**B**–**I**) Box-and-whisker plots (median, Q1, Q3, min-max) showing the expansion of total (**B**), IgM (**C**), IgG1–IgG4 (**D**–**G**), and IgA1 and IgA2 (**H** and **I**) plasma cells after challenge expressed as ratio compared to baseline (**B**–**I**: *n* = 14). Shown are medians and range. Dashed line indicates a ratio of 1.0 (baseline value). Baseline cell counts for the entire cohort, median (min-max) in cells/μL are indicated below each graph and merely added as an indication of frequency of the cell population. Longitudinal changes were evaluated using Wilcoxon’s matched-pairs signed-rank test on ratio versus baseline. Correction for multiple testing was performed using Bonferroni’s post hoc test. In cases of significant differences in ratio compared with baseline, the median increase in cells/μL is indicated on top of the bar. D, days after challenge. ^a^Participant ID.12 showed strongly elevated total and IgM plasma cell counts at baseline, which were approximately 5-fold (total plasma cells) and 25-fold (IgM plasma cells) higher compared with counts measured on D28 and D56. Therefore, this participant was excluded in panels **B**–**I**.

Upon challenge, fluctuations in the naive and memory B cell compartment were limited and not consistent between participants (Figure 5A, cell counts presented in Supplemental Table 1). However, plasma cells showed a trend toward increased cell numbers on D11–D14 after challenge (ratio vs. baseline); this expansion was observed in all isotypes (Figure 4, B–I, and Supplemental Figure 5A) and more prominent in participants inoculated with 1 × 10^5^ CFU (Supplemental Figure 5B, trend). Of note, one participant (ID.12) showed strongly elevated (IgM) plasma cell counts at baseline. Therefore, data from this participant were not included in Figure 5, B–I. No consistent changes were observed in the distribution of maturation stages of total plasma cells, which were primarily defined by the loss of CD20 and gain of CD138 markers (Supplemental Figure 4D).

**Figure 6:**
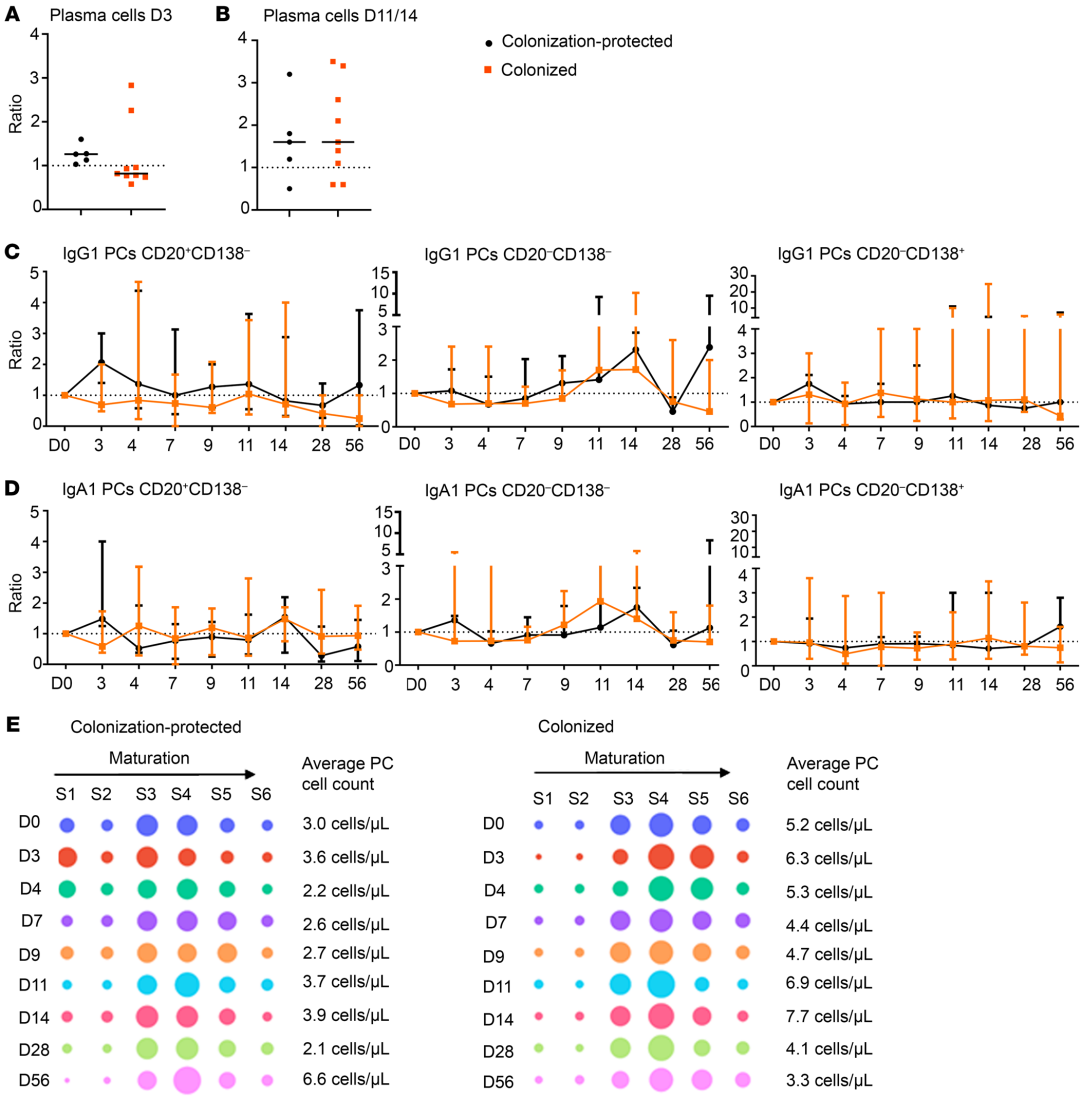
Different kinetics in the plasma cell compartment of colonized and colonization-protected participants. Note: Due to different normalization of plasma cells in participant ID.12, this participant was not included in the analysis of total plasma cells (panels **A**, **B**, and **E**; *n* = 14). (**A**) Plasma cell expansion on D3 after challenge, expressed as ratio compared to baseline. (**B**) Plasma cell expansion on D11–D14 after challenge expressed as ratio compared to baseline (of D11 and D14, the day of maximum expansion was used for each participant). *n* = 14. (**C** and **D**) Longitudinal changes in IgG1 (**C**) and IgA1 (**D**) plasma cell (PC) maturation stages expressed as ratio and min-max compared with baseline. *n* = 15. Dashed line indicates a ratio of 1.0 (baseline value). *n* = 15. Statistical test performed for longitudinal analysis: Wilcoxon’s matched-pairs signed-rank test on ratio of baseline. Correction for multiple testing was performed using Bonferroni’s post hoc test. Statistical test for comparison between groups per time point: Mann-Whitney *U* test on ratio of baseline. (**E**) Distribution of total plasma cells over 6 different maturation stages (S1–S6), expressed as percentage of total plasma cell population. Average plasma cell counts are indicated on the right side of each plot.

Next, we grouped the participants based on colonization status. Here, we observed an expansion of plasma cells on D3, which was most prominent in colonization-protected participants, but found in 7 participants in total (Figure 6A, trend). Among all plasma cell subsets, expansion on D3 seemed slightly more prominent for IgG1 (trend), IgG4 (trend), and IgA1 (trend). Plasma cell expansion on D11–D14 seemed unrelated to colonization status (Figure 6B). Next, we investigated the maturation patterns of IgG1 and IgA1 plasma cells in colonized and colonization-protected participants (Figure 6, C and D). We observed that in colonization-protected participants the expansion of IgG1 on D3 after challenge mostly consisted of the least and most mature IgG1 plasma cells, whereas for IgA1 this expansion consisted of the least and intermediate mature IgA1 plasma cells. For IgG4 plasma cells, cell counts were generally too low to reliably monitor all 3 plasma cell maturation stages. When assessing the distribution of total plasma cells over different maturation stages, we found a relative increase in more immature plasma cells on D3 and D4 after challenge in colonization-protected participants, but not in colonized participants (Figure 6C).

Then, we divided participants based on colonization density. Aside from colonization-protected participants, the plasma cell expansion on D3 was observed in 1 low- and 1 high-density-colonized participant (Supplemental Figure 5A). Expansion of plasma cells on D11–D14 after challenge was not specifically related to colonization density (Supplemental Figure 5B). Upon inspection of IgG1 and IgA1 plasma cells, we found that on D3 after challenge, colonization-protected participants had significantly higher numbers of the least mature IgA1 plasma cells, as compared with low-density-colonized participants. Moreover, both high-density-colonized and colonization-protected participants showed an increase in intermediate mature IgG1 and IgA1 plasma cells on D11–D14 after challenge (Supplemental Figure 5, C and D; trend). When assessing the distribution of total plasma cells over different maturation stages, the differences between low-density-colonized and colonization-protected participants were most prominent at early time points (D0, D3, and D4) (Supplemental Figure 5E).

As the expansion of plasma cells on D3 after challenge seemed more prominent in colonization-protected participants, we correlated the plasma cell expansion on D3 with the maximum CFU load and the CFU load on each day after challenge until the first day of azithromycin treatment. No correlation was found between the plasma cell expansion and the maximum CFU load. Instead, we found a negative correlation between plasma cell expansion on D3 and CFU load on D7 (Spearman’s *r* = –0.5773, *P* = 0.0269) and CFU load on D9 (Spearman’s *r* = –0.6215, *P* = 0.0157). Plasma cell expansion on D3 did not correlate with CFU load on D11 or D14. Moreover, as T cell help is required for B cell activation, we correlated the plasma cell expansion on D3 with Th cell expansion on D1 and Tfh expansion on D3, but no correlation was observed. Lastly, we correlated the plasma cell expansion on D3 with the anti-PT IgG levels at baseline. Although during initial screening anti-PT IgG levels greater than 20 IU/mL were used as an exclusion criterion, anti-PT IgG levels determined at actual baseline (second screening) in the participants ranged from 0.2–22.2 IU/mL, possibly suggesting a more recent *B*. *pertussis* encounter in some individuals. Indeed, we found a positive correlation (Spearman’s *r* = 0.6452, *P* = 0.01111) between these 2 values, indicating that cellular and humoral responses parallel each other.

Thus, within the B cell compartment, we observed most changes within the IgG and IgA plasma cell subsets, yet all were subtle and should therefore be confirmed in a larger study cohort. An early expansion of plasma cells on D3 after challenge was primarily observed in participants protected against colonization, whereas plasma cells on D11–D14 after challenge expanded irrespective of colonization status. A negative correlation was observed between CFU load and plasma cell expansion on D7 and D9 after challenge. Lastly, maturation of the IgG1 and IgA1 plasma cells was observed to some extent, especially toward an intermediate mature phenotype on D11–D14 after challenge.

### Plasma cell expansion is a predictor of seroconversion.

**Figure 7:**
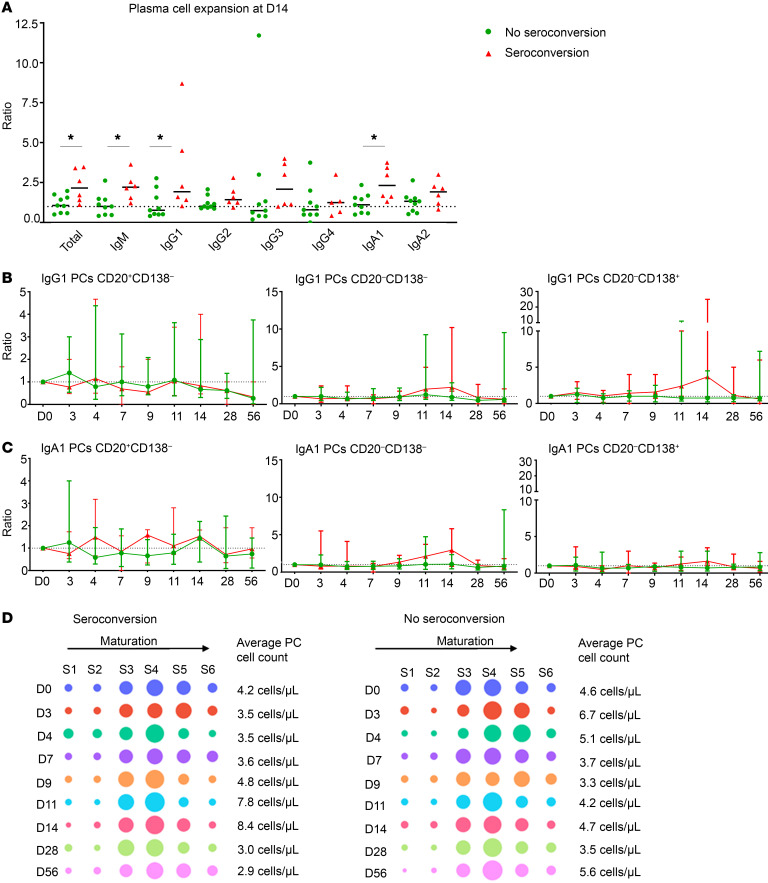
Different plasma cell kinetics in participants that did or did not seroconvert. (**A**) Expansion of plasma cell numbers on D14 after challenge. Expansion is expressed as ratio compared to baseline. *n* = 15. (**B** and **C**) Longitudinal changes in IgG1 (**B**) and IgA1 (**C**) plasma cell maturation stages expressed as ratio and min-max compared with baseline. *n* = 15. Dashed line indicates a ratio of 1.0 (baseline value). *n* = 15. Statistical test performed for longitudinal analysis: Wilcoxon’s matched-pairs signed-rank test on ratio of baseline. Correction for multiple testing was performed using Bonferroni’s post hoc test. Statistical test for comparison between groups per time point: Mann-Whitney *U* test on ratio of baseline. *P < 0.05 (longitudinal change). (**D**) Distribution of total plasma cells over 6 different maturation stages (S1–S6), expressed as percentage of total plasma cell population. Average plasma cell counts are indicated on the right side of each plot.

Ag-specific serum IgG levels are routinely used as a readout for vaccine efficacy and/or protective immunity; for example, anti-capsular IgG serum levels correlate with protection against *Streptococcus pneumoniae* (27). Therefore, we investigated cellular changes associated with seroconversion. Here, we found that all participants who seroconverted (1 low-density- and 5 high-density-colonized participants) had a more prominent expansion of total plasma cells on D14 after challenge compared with nonseroconverting participants (*P* < 0.05) (Figure 7A). This expansion was clearest in IgM, IgG1, and IgA1 plasma cells (*P* < 0.05). Yet, only a trend toward an increased number of intermediate (D14) and most mature (D11 and D14) IgG1 plasma cells was observed in participants who seroconverted compared with nonseroconverting participants (Figure 7B). For IgA1 plasma cells, a trend toward higher intermediate mature plasma cell counts was found in seroconverting participants on D11 and D14 (Figure 7C). When observing the distribution in maturation stages in the total plasma cell compartment, we observed a slight increase in more mature plasma cells in nonseroconverting participants on D3–D4 after challenge (Figure 7D).

Evaluation of other immune cell subsets did not reveal any additional cellular changes specific for seroconversion (cell counts presented in Supplemental Table 1). Lastly, we investigated the correlation between maximum colonization density in CFU during the study and the absolute increase in serum IgG (D28 – D0) directed against PT, FHA, Prn, and Fim2/3. We found a positive correlation for anti-PT IgG (Spearman’s *r* = 0.6486, *P* = 0.0109) and anti-FHA (Spearman’s *r* = 0.5684, *P* = 0.0296), but not for anti-Prn or anti-Fim2/3 IgG.

### Baseline B. pertussis–specific serum IgG levels correlate negatively with maximum CFU count.

It is known that an individual’s baseline immune status can impact the individual’s immune response. Therefore, we correlated the number of plasma cells at baseline and the *B*. *pertussis*–specific IgG serum levels with the maximum CFU load. Although anti-PT IgG serum levels greater than 20 IU/mL correlated with expansion of plasma cells on D3, they did not correlate with maximum CFU count (Spearman’s *r* = –0.2496, *P* = 0.3655). However, we found a negative correlation between *B*. *pertussis*–specific IgG levels and maximum CFU counts for anti-FHA (Spearman’s *r* = –0.5292, *P* = 0.0447), anti-Prn (Spearman’s *r* = –0.6170, *P* = 0.0166), and anti-Fim2/3 (Spearman’s *r* = –0.5210, *P* = 0.0487), possibly indicating preexisting immunity in several participants. No correlations were found between baseline plasma cell numbers and the maximum CFU load.

### Most informative time points and cell populations associated with protection against colonization.

**Figure 8:**
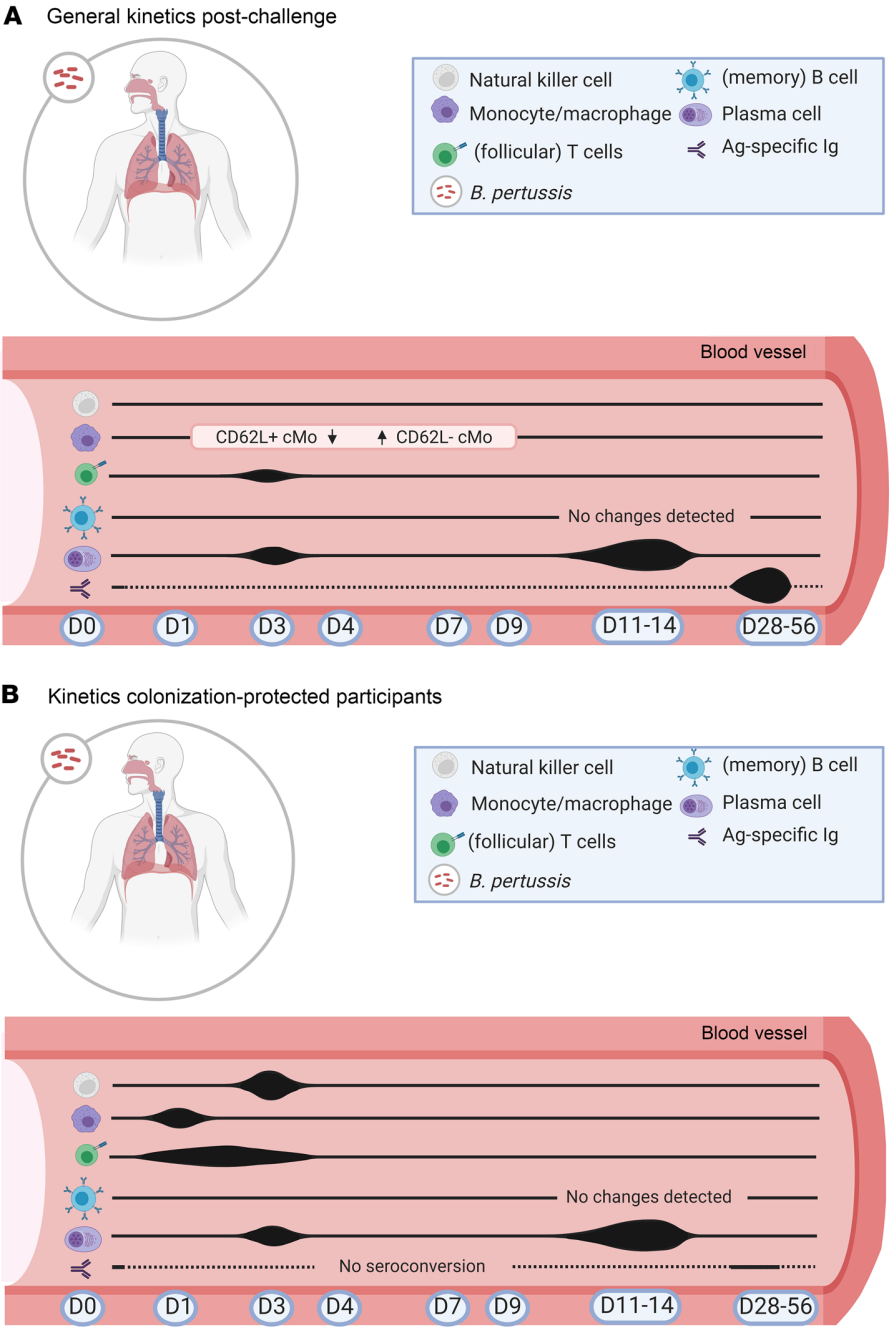
Overview of the most informative time points and cell populations after bacterial challenge. (**A**) General cellular kinetics after challenge irrespective of clinical readout. (**B**) Cellular kinetics unique to participants protected against colonization, possibly indicating preexisting immunity in these participants.

This study identified the immune cell populations and sampling time points that are the most informative for follow-up studies. Especially in the early days after bacterial challenge (D1, D3, and D4), changes in the innate immune compartment were detected. Changes in the adaptive immune response can be monitored at various time points during the 2 weeks after challenge, with D1, D3, D11, and D14 being most informative. The most informative time points and cell populations to follow up on after bacterial challenge are summarized in Figure 8A (general kinetics after challenge) and Figure 8B (kinetics specific for colonization-protected participants).

## Discussion

This study describes cellular immune responses following bacterial challenge and evaluates which features of this response correlate with protection against colonization and/or seroconversion status. First, we evaluated the general immune cell kinetics after challenge. Next, we assessed differences in immune signature based on colonization, density, or seroconversion status. In the early days (D1, D3, and D4) after bacterial challenge, multiple cellular changes were observed in the blood, especially in the monocytic compartment, Tfh cells, and plasma cells. Kinetics of these subsets differed between colonized and colonization-protected participants, and surprisingly, the most prominent differences were found between low-density-colonized and colonization-protected participants. Interestingly, the D3 plasma cell expansion negatively correlated with CFU load on D7 and D9 after challenge. Later, on D11–D14 after challenge, a trend toward expansion of various plasma cell subsets was observed. Although this exploratory study consisted of only 15 wP-primed participants, which were clustered based on their colonization status (leading to low statistical power), we detected consistent kinetics related to clinical readouts (colonization and seroconversion status). These findings should be further confirmed in a larger study cohort.

An important aspect of vaccine evaluation and improvement is in understanding the underlying immune response. To our knowledge, cellular immunity against *B*. *pertussis* colonization has never been studies in humans, but comparison can be made with vaccination studies, where protection against pertussis is achieved through intramuscular vaccination. There are multiple studies describing antibody responses, and some studies also describe cellular responses induced by intramuscular vaccination against pertussis (19, 23–25, 28). The major differences between current *B*. *pertussis* vaccines and natural infection are the location of Ag encounter (intramuscular vs. mucosal) and the variety and concentration of Ags (aP vaccines contain purified *B*. *pertussis* Ags usually combined with compounds from other bacteria/viruses like tetanus, diphtheria, polio). Although *B*. *pertussis* vaccines confer protection against disease, they do not seem to prevent colonization and transmission of the bacterium (3, 29, 30). Therefore, it is of critical importance to understand what is required to prevent colonization, translocation (the movement of bacteria from respiratory surfaces into the underlying, otherwise sterile tissues), and transmission of *B*. *pertussis*. The benefit of reduced carriage has already been suggested for other nasopharyngeal microbe–related infectious diseases, such as meningococcal disease and *Haemophilus influenzae* type b (31, 32). There, intramuscular vaccination resulted in reduced carriage, which was suggested to lead to increased herd immunity/protection because of interrupted transmission (of note, for meningococcal disease, this was shown for polysaccharide-conjugated vaccine). This interrupted transmission was most likely caused by increased mucosal immunity. Indeed, vaccination against meningococcal disease caused by serogroup A and C not only reduced carriage, but also led to an increase in IgG and IgA after booster vaccination (probably as a result of exudation of plasma on the mucosal surfaces), leading the authors to conclude that mucosal immunity was boosted (33).

In 2 previous vaccination studies, we explored the cellular responses to intramuscular aP booster vaccination in various cohorts using highly similar methods (aP-primed children, aP- or wP-primed adolescents, wP-primed young adults, and wP-primed older adults) (23, 34). Both studies pointed toward the D7 plasma cell expansion and maturation as the major cellular change after vaccination. One of these studies also investigated the innate immune cell and T cell kinetics in 10 wP-primed participants who received an aP booster (23). There, we observed “late” monocyte kinetics on D3 after vaccination (mostly iMos and ncMos) in approximately half of the participants. No consistent changes were observed after vaccination in the numbers or composition of the CD4^+^ T cell, CD8^+^ T cell, or NK cell compartment. Comparing these studies with the here-presented controlled infection study, we found clear differences between the kinetics after challenge and after vaccination, which are discussed in more detail below (cell kinetics after challenge and vaccination can also be found in Supplemental Table 1). Interestingly, several of the findings in this challenge model were in line with a recent study evaluating the immune responses after intranasal vaccination with an attenuated *B*. *pertussis* strain (10).

Of all evaluated innate immune cells, monocyte subsets hold promise for future analysis. The decrease in CD62L^+^ cMos and increase in CD62L^–^ cMos implies activation and maturation of the cMos. This activation was observed irrespective of clinical outcome. However, the early expansion of (especially) CD36^–^ ncMos was found exclusively in participants protected against colonization. Currently, information about the exact role of CD36^–^ ncMos is scarce. However, part of the CD36^–^ ncMos are SLAN^+^ ncMos. SLAN^+^ ncMos were recently reviewed by Ahmad et al. (35) and are known to be circulating and tissue myeloid cells with high plasticity and a proinflammatory function.

In our previously published vaccination study, we did not observe the activation of cMos or the expansion of CD36^–^ ncMos, although an overall expansion of iMos and ncMos was found (23). This may be explained by the lack of early time points evaluated after vaccination or by the different mode of Ag encounter. Recent work on intranasal vaccination with a live attenuated *B*. *pertussis* (BPZE1) did not observe consistent changes or maturation in cMos, iMos, or ncMos (10). This may be attributed to differences in methodology, such as depth of evaluation, (number of) evaluated time points, or the use of an attenuated *B*. *pertussis* strain. Interestingly, the authors reported the production of several proinflammatory cytokines by purified monocytes upon stimulation with BPZE1, implying that upon contact with *B*. *pertussis*, the monocytes start a local inflammatory response.

The expansion of NK cells seemed to have an inverse relationship with colonization density. This expansion was not observed upon booster vaccination with aP, as was the case with intranasal study reports on NK cell kinetics (10, 23). Animal studies have shown a role for NK cells in *B*. *pertussis* infection, where they are thought to be the initial source of IFN-γ (reviewed by Higgs et al.; ref. 36). The production of IFN-γ is essential for containment of the infection and promotion of the Th1 response. Although we did not monitor IFN-γ secretion, we did find an early, specific NK cell signature (expansion) in the colonization-protected participants.

Interestingly, we found that low-density-colonized donors showed more prominent innate immune cell kinetics compared with high-density-colonized participants. A possible explanation might be the presence of some preexisting immunological memory, yet not as high as in participants protected against colonization. This preexisting memory might result in a more targeted innate immune response (influenced by preexisting memory cells or antibodies) and faster control of bacterial growth as compared with donors without any noteworthy preexisting immunological memory.

Within the T cell compartment, we found an increase in Tfh cells on D3 after challenge in participants protected against colonization. This was not observed after aP booster vaccination, yet an expansion of activated Th1-like Tfh cells was observed from D4 after intranasal vaccination (with BPZE1) (10). Activation and/or expansion of Tfh cells can support the formation of germinal centers and thus initiate humoral responses, leading to the generation of (protective) antibodies. Moreover, we found increased numbers of circulating Th cells of the Th1/Th17, Th22, and CXCR3^–^CCR4^–^CCR6^+^CCR10^^–^^ phenotype on D1 after challenge in donors protected against colonization (and as a trend on D3). This was not observed after an aP booster vaccination, and is much earlier than the T cell readout used in the evaluation of BPZE1 vaccination, where a Th1 response was found in stimulated PBMCs on D28 after vaccination (10). A potential role of Th22 and Th cells of the CXCR3^–^CCR4^–^CCR6^+^CCR10^–^ phenotype in the context of *B*. *pertussis* infection has to the best of our knowledge not been described and, upon confirmation in a larger cohort, should be further investigated.

In this study, we observed a trend toward expansion of several IgG and IgA plasma cell subsets on D3 (mostly in colonization-protected participants) and on D11–D14 after challenge. This differs from postvaccination kinetics, where we observed a prominent and homogeneous expansion and maturation of (primarily IgG1) plasma cells on D7 after vaccination. This difference may be explained by the manner of Ag encounter. During the booster vaccination, a high dose of Ag (plus adjuvant) is injected into the muscle, and thus all participants will be exposed to the Ag at the same time (D0), resulting in a synchronized induction of the immune response in vaccinated donors. In intranasal bacterial challenge, all participants are challenged with *B*. *pertussis* on D0, but there may be differences in bacterial growth between participants. Therefore, the plasma cell peak may differ in timing between participants and thus not be as homogeneous as after receiving a booster vaccination. Like in the postchallenge data, the expansion of (total) plasma blasts after intranasal vaccination with BPZE1 peaked on D14 (10). Interestingly, in that same set of data, in 2 of 12 participants an expansion of plasma blasts/plasma cells seemed present already on D4 after vaccination, although the authors did not comment on this (10).

The observed IgG response is most likely the result of previous vaccine-induced immunity against *B*. *pertussis* and natural boosting (all participants were wP-primed during childhood and thus not *B*. *pertussis* naive), whereas the IgA response can be mainly attributed to previous natural pathogen encounters. Of note, once generated, both IgG and IgA memory B cells may be triggered by vaccination and/or infection. As natural infection occurs via the respiratory tract, local mucosal immunity (i.e., secretory IgA antibodies and IgG antibodies via exudated plasma) is of importance in the rapid neutralization of *B*. *pertussis* upon infection. Increased mucosal immunity should be an important aspect for future *B*. *pertussis* vaccine candidates, as this may reduce transmission. We believe that before this can be evaluated, the cellular responses preventing colonization and transmission should be understood. Of course, the question is to what extent the circulating IgA plasma cells represent mucosal IgA responses. Additional analysis of surface or intracellular markers may give insight in the destination of these plasma cells, e.g., the use of markers that allow differentiation between circulating and mucosa-oriented plasma cells, such as adhesion molecule β7 integrin, J-chain, or CCR10 (37–39), may help to understand the destination of the expanding plasma cells. Expression of the J-chain would be of special interest for the peak of plasma cells that was observed on D3 after challenge in the participants who were protected against colonization, as expression of mucosal homing markers may be lost when these cells are recruited from mucosal layers into the circulation. Moreover, repertoire studies comparing the (Ag-specific) B cell receptor (BCR) repertoire between B cells at baseline, the plasma cells on D3 and on D11–D14, and memory B cells at later time points may yield valuable insights in the relationship of these cells.

In this study, all participants were inoculated with predefined doses of the well-characterized *B*. *pertussis* strain B1917, which is a fully genotyped representative of current European isolates (40). We acknowledge that a controlled experimental infection (aiming for colonization) is not the same as a natural infection model. The mode of infection, the dose, and circumstances are designed to be standardized and this might be a limitation to translating these findings to natural pertussis infection. Nevertheless, we clearly observed shared kinetics within the total cohort, and when stratifying based on colonization status.

Not all participants who were colonized showed signs of seroconversion. Perhaps translocation of *B*. *pertussis* is required to initiate seroconversion. In that case, although these participants were colonized, the bacteria did not fully translocate into the tissue in all participants, leading to seroconversion only in the participants in whom bacteria had translocated. It would be interesting to compare the kinetics of antibodies in the mucosal lining fluid of the nose in participants who did and did not seroconvert and their colonization density. Additionally, it may be of interest to study whether the mucosal antibodies and the early plasma cell peak are associated with each other (e.g., by comparing BCRs). Lastly, it may be interesting to investigate whether participants can transmit *B*. *pertussis* to others, as this would confirm that asymptomatic infection results in transmission of *B*. *pertussis*. Such a finding would imply that the presence/absence of symptoms and the serological status may not correctly reflect carriage/transmission state, and thus that serological surveillance studies, although valuable, may still underestimate the number of *B*. *pertussis* infections.

In this cellular immune monitoring study, we evaluated the overall immune cell kinetics in the blood. Immune cell populations in the blood have been well characterized in several studies and their phenotype and quantities can be relatively easily compared between studies. One of the limitations of this approach is that we may not capture part of the local responses occurring at the pathogen encounter site, i.e., within the (mucosal) tissues. We acknowledge that such cells would be a valuable source of information and their evaluation could be included in follow-up studies. However, due to the burdensome nature of this type of sample collection, it is unlikely that such an analysis would be included in any routine type of analysis. Another possibility to dissect cellular changes specific to colonization/pathogen encounter is to focus on changes in pathogen-specific cells of the adaptive immune system, i.e., B cells and T cells recognizing *B*. *pertussis*. This could be easier to include in daily practice.

Here, we monitored the overall (non–Ag-specific) kinetics of immune cells. We previously showed that there is a fair correlation between Ag-specific ELISpot data and plasma cell flow cytometry data in postvaccination settings (41). Although no ELISpot experiments were performed in the here-reported study, we did observe an increased number of (total and more mature) plasma cells on D14 after challenge in donors showing seroconversion. The latter is in line with the ELISpot data that was previously reported on the total cohort used in the dose-finding study of this human challenge model (12). There, increased numbers of Ag-specific Ig–producing plasma cells were observed in multiple colonized participants (*n* = 16) on D14 after challenge, but not in participants protected against colonization (*n* = 9). In both studies, seroconversion was only observed in colonized participants. Given the relationship between Ag-specific Ig–producing plasma cells and the serum Ig levels, the participants showing increased numbers of Ag-specific Ig–producing plasma cells are likely the donors with seroconversion. Aside from ELISpot, an Ag-specific approach combined with mucosa-tracing markers may be informative, possibly helping us to find (surrogate) biomarkers of protection in the blood. An Ag-specific approach would be especially relevant to monitor kinetics of specific memory B and T cells.

One remaining question is whether the early response observed in participants protected against colonization is indeed protective, and to what extent this information can be used in future studies. To confirm whether a protective response is observed in participants, a rechallenge would indeed be required (e.g., an infection-reinfection cohort). As we know that natural infection gives the best protection (compared with wP and aP vaccination) (3, 6), it may be that this controlled infection leads to a similar level of protection. Based on our data, we hypothesize that the early expansion of CD36^–^ ncMos, NK cells, Tfh cells, and (possibly) plasma cells in the colonization-protected participants may be associated with an efficient mucosal immune response, preventing colonization. When rechallenging the same group of individuals, we would expect a higher number of participants to be protected against colonization, and thus to show this same early cellular response.

To the best of our knowledge, we are the first to investigate such in-depth cellular immune responses in a *B*. *pertussis* human challenge model. Here, we report which cell populations and time points can yield valuable information about the colonization status and how this compares to vaccine-induced cellular kinetics. Because of the low incidence of pertussis and the difficulty in diagnosing it early, there is a lack of information on cellular responses upon *B*. *pertussis* infection in humans. Increased insight, especially in the protective immune signature, is crucial to the development of novel pertussis vaccines, which should aim to prevent colonization and transmission of *B*. *pertussis*. This study provides insights into the cellular responses in individuals with varying degrees of colonization upon controlled bacterial challenge. As we only investigated 15 participants, these findings should be corroborated in future studies. When fully established, this human challenge model can not only be used to study *B*. *pertussis* infection, colonization, translocation, transmission, and/or shedding in challenged individuals, but also to dissect the induced immune responses: early versus late responses, mucosal versus systemic responses, and protective versus nonprotective responses. More importantly, controlled bacterial challenge could be an important step to evaluate the efficacy of novel *B*. *pertussis* vaccine candidates with respect to protection against colonization and transmission.

## Methods

### Inclusion criteria and sample collection.

Peripheral blood (PB) samples were collected during a dose-finding controlled infection study using *B*. *pertussis* (ClinicalTrials.gov NCT03751514, ethical committee reference 17/SC/0006), which aimed to determine the dose required to colonize at least 70% of participants, and was reported by De Graaf et al. (11, 12). Participants (wP-primed) were eligible for the study when they were healthy, aged 18 to 45 years, available for the 16-day admission period and scheduled visits, vaccinated against *B*. *pertussis* at least 5 years before enrollment, did not use antibiotics within 4 weeks before enrollment in the study, had no contraindication to azithromycin (administered on D14, D15, and D16), and had no contact with individuals vulnerable to pertussis (full eligibility criteria were reported previously; ref. 12). Participants with recent exposure to *B*. *pertussis* and increased chance of protective immunity against *B*. *pertussis*, defined as baseline serum anti-PT IgG greater than 20 IU/mL (determined by ELISA), were excluded. Participants were admitted to the National Health Institute for Health Research Clinical Research Facility in Southampton, UK and challenged by receiving a nasal inoculum of 1 mL (0.5 mL per nostril) containing *B*. *pertussis* strain B1917, which is a fully genotyped representative of current European isolates (40). A full description of inoculation procedure is described in the study protocol (11). A total of 34 participants were challenged with various doses of *B*. *pertussis*. This paper describes 15 healthy participants (*n* = 15; M/F ratio, 8:7; age range, 18–43 years old) who were challenged intranasally with 1 × 10^4^ (*n* = 5) or 1 × 10^5^ (*n* = 10) CFU of *B*. *pertussis* strain B1917.

Colonization was assessed by culture of nasal wash samples on *B*. *pertussis*–specific media on D4, D5, D7, D9, D11, D14, D15, and D16 after challenge (12). Colonization was defined as any *B*. *pertussis–*positive culture at any time point after challenge. Colonization density was stratified into 3 categories: colonization-protected (0 CFU/mL), low-density colonized (<1000 CFU/mL), and high-density colonized (>1000 CFU/mL) at any time point.

PB was collected in EDTA blood collection tubes on D0, D1, D3, D4, D7, D9, D11, D14, D28, and D56 after challenge. PB was taken early in the morning and transported to Leiden University Medical Center (LUMC), the Netherlands, where flow cytometric evaluation was performed within 12 hours after donation. In 2 participants (ID.08 and ID.13), the expression of CD45RA on lymphocytes was found to be unusual, most likely caused by a CD45RA-related polymorphism (21), hampering reliable identification of most T cell subsets in these 2 participants. Aside from the anti-PT ELISA used for initial screening, serum IgG against PT, Prn, FHA, and Fim2/3 was measured at baseline and on D28 using multiplex immune assay (42) at the National Institute for Public Health and the Environment (RIVM, the Netherlands). In this study, seroconversion is defined as a greater than 2-fold increase in anti-PT IgG compared with baseline.

### Blood processing and staining — immune monitoring panels and absolute count determination.

All PB samples were subjected to high-throughput EuroFlow-based flow cytometric immunophenotyping with 4 multicolor immune monitoring panels (or their direct prototypes). These panels allow monitoring the kinetics of over 250 circulating immune cell subsets. In short, the B cell and plasma cell tube (BIGH) allows identification of up to 115 B and plasma cell subsets that are distinguished based on expressed Ig subclasses and their maturation stage–associated phenotype (41, 43, 44). The CD4^+^ T cell panel (CD4T) allows identification of more than 89 CD4^+^ T cell subsets with different functionalities and maturation stages (20). The CD8^+^ cytotoxic T cell panel (CYTOX) allows identification of up to 50 subsets within the CD8^+^ T cell and NK cell compartment (43). Lastly, the DC-monocyte panel (DC-Monocyte) allows identification of up to 19 different (sub)populations within the myeloid compartment, including subsets of monocytes and DCs (45).

All blood samples were processed according to the EuroFlow standard operating protocol (SOP) as reported previously (protocols available at www.EuroFlow.org) (23, 41). Two adjustments of the previously reported methods concerned the addition of surface marker CD45 to the BIGH, CD4T, and CYTOX panels, and cytoplasmic staining with an anti-CD154 antibody in the CD4T and the CYTOX panel. The latter resulted in an additional intracellular staining step for the CYTOX panel.

In short, for CD4T and CYTOX panels, 100 μL PB was used for membrane and intracellular staining using the Fix and Perm Kit (Sanbio). For the BIGH and DC-Monocyte panel, high cell numbers were required, and thus a bulk lysis was performed on whole blood, and then 10 million cells were stained for membrane markers. For the BIGH panel, this was followed by intracellular staining for Igs (again, using the Fix and Perm Kit). For the DC-Monocyte panel, the surface staining was followed by a 10-minute incubation with FACS lysing solution (BD Biosciences), washed, and acquired on the flow cytometer.

Absolute cell counts were determined with the use of Perfect-Count Microspheres (Cytognos) according to the EuroFlow SOP (www.EuroFlow.org). In short, exactly 50 μL of PB was stained with anti-CD19–BV786 (BD Biosciences, clone SJ25C1, catalog 563326; 4 μL), anti-CD3–APC (BD Biosciences, clone SK7, catalog 340440; 2.5 μL), and anti-CD45–OC515 (Cytognos, clone GA90, catalog CYT-45OC; 5 μL), and samples were incubated for 30 minutes in the dark. Next, 500 μL of NH_4_Cl (in-house solution) was added, and after a 10-minute incubation exactly 50 μL of Perfect-Count Microspheres (CYT-PCM-50-R, Cytognos) was added. Samples were acquired immediately afterwards. This procedure allowed for accurate assessment of absolute cell counts of leukocytes, lymphocytes, B, T, and NK cells.

All samples were measured on an LSR Fortessa 4L or on an LSR Fortessa X-20 4L (BD Biosciences). Flow cytometer performance was assessed daily according to the EuroFlow guidelines, as previously described (46, 47). Staining with all antibody panels was performed at every time point, with the exception of D1 after challenge, in which the BIGH panel was not applied. An overview of all used antibodies and the original publications/sources mentioning these antibody panels can be found in Supplemental Tables 2–5. Phenotypic definitions of all populations can be found in Supplemental Tables 6–9.

### Data analysis.

All data were analyzed manually in Infinicyt Software (v2.0, Cytognos) according to the EuroFlow gating strategies (20, 41, 43, 45). When comparing cellular kinetics between groups with different clinical readout, first all longitudinal data were evaluated, and the most prominent changes are shown in the figures. Plasma cell maturation stages were defined using the maturation pathway tool in the Infinicyt software, as previously described (23). Cell counts at follow-up visits were normalized to the corresponding baseline (D0) values for each donor individually. To increase data transparency, all population cell counts can be found in Supplemental Table 1. This table also includes cell numbers of the major and most relevant cell populations identified in the aP booster study that is referred to multiple times in the Discussion section of this paper (23).

### Statistics.

Differences in cell counts between multiple (>2) conditions were assessed with Kruskal-Wallis 1-way ANOVA, followed by Dunn’s test. Differences between cell counts/ratios in 2 tested conditions were assessed with the Mann-Whitney *U* test. A *P* value of less than 0.05 was considered statistically significant. Longitudinal changes were evaluated with Wilcoxon’s matched-pairs signed-rank test, and corrected for multiple testing using Bonferroni’s post hoc test. GraphPad Prism 8.0 software was used for statistical tests. Correlations between 2 parameters were assessed using Spearman’s ranking correlation.

### Study approval.

This study was conducted in accordance with the provisions of the Declaration of Helsinki (1996) and the International Conference on Harmonization Guidelines for Good Clinical Practice. This study is registered with ClinicalTrials.gov (NCT03751514; ethical committee reference 17/SC/0006) and was approved by the South Central – Oxford A Research Ethics Committee. Written informed consent was obtained from every individual before participation.

## Author contributions

CT, RCR, JJMVD, and MAB conceived the study. HDG and RCR developed methodology. AMD, HDG, CT, RJG, BDM, MI, and MAB conducted formal analyses. AMD, HDG, CT, RJG, BDM, MI, ARH, and MAB carried out the investigation. AMD and MAB wrote the original draft of the manuscript, which was reviewed and edited by AMD, HDG, CT, RJG, BDM, MI, ARH, RCR, JJMVD, and MAB. AMD generated figures. RCR, JJMVD, and MAB supervised the study. HDG, RCR, JJMVD, and MAB provided project administration. RCR and JJMVD acquired funding. All authors have read and agreed to the published version of the manuscript. AMD and HDG both contributed substantially to this manuscript and share first author position. The authorship order between shared first authors was assigned alphabetically.

## Supplementary Material

Supplemental data

ICMJE disclosure forms

Supplemental table 1

## Figures and Tables

**Table 1:**
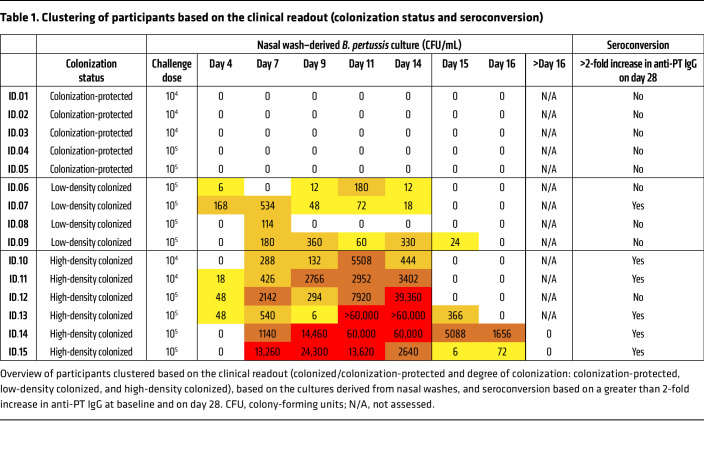
Clustering of participants based on the clinical readout (colonization status and seroconversion)

## Version 1 01/17/2023

In-Press Preview

## Version 2 03/01/2023

Electronic publication
