# Supplementary material for: Distinct early cellular kinetics in participants protected against colonization upon Bordetella pertussis challenge
Source: J Clin Invest. 2023 Mar 1;133(5):e163121. doi: 10.1172/JCI163121 (PMC9974097; doi:10.1172/JCI163121)
Supplement: Supplemental data [file jci-133-163121-s151.pdf]

Supplemental Tables, Figures, and Acknowledgements

Supplemental Table 1. This supplemental Table can be found as a separate spreadsheet added to this manuscript.

Supplemental Table 2. Overview of all antibodies used in the EuroFlow PERISCOPE BIG panel. Official references for this panel: Blanco E, Pérez-Andrés M, Arriba-Méndez S, et al. Age-associated distribution of normal B-cell and plasma cell subsets in peripheral blood. *JACI* 2018;141(6):2208-2219. e2216. Diks AM, Versteegen P, Teodosio C, et al. Age and Primary Vaccination Background Influence the Plasma Cell Response to Pertussis Booster Vaccination. *Vaccines.* 2022;10(2):136. Patent filed by Van Dongen et al. Means and Methods for Multiparameter Cytometry-Based Leukocyte Subsetting. P119646NL00 (2019). PCT/NL2020/050688, priority date 5 November 2019.

Marker	Fluorochrome	Clone	Source	Cat. number	Volume (µL)	Membrane	Intracellular
CD27	BV421	M-T271	BD	562513	2	All added to membrane staining	
IgM	BV510	MHM-88	BioLegend	314522	2		X
CD62L	BV605	DREG-56	BioLegend	304833	5		
CD24	BV650	ML5	BD	563720	5		
CD21	BV711	B-Ly4	BD	563163	5		
CD19	BV786	SJ25C1	BD	563163	4		
Subclasses cocktail	-	-	Cytognos	CYT-IGS-1	25		X
IgD	FITC	IA6-2	BioLegend	348205	1.25		X
CD20	PE CF594	2H7	BD	562295	2.5 (1:10 dil)		
CD138	PE-Cy7	MI15	BioLegend	356513	5		
CD5	PE-Cy7	LIF7F12	BD	348810	6		
IgD	APC	IA6-2	BD	561303	4		X
CD45	AF700	HI30	BD	560566	10		
CD38	APC H7	HB7	BD	656646	3		

Supplemental Table 3. Overview of all antibodies used in the EuroFlow PERISCOPE CD4 T-cell panel. Official reference for this panel: Botafogo V, Pérez-Andres M, Jara-Acevedo M, et al. Age distribution of multiple functionally relevant subsets of CD4+ T cells in human blood using a standardized and validated 14-color EuroFlow immune monitoring tube. *Frontiers in immunology.* 2020;11:166. Patent filed by Van Dongen et al. Means and Methods for Multiparameter Cytometry-Based Leukocyte Subsetting. P119646NL00 (2019). PCT/NL2020/050688, priority date 5 November 2019.

Marker	Fluorochrome	Clone	Source	Cat. number	Volume (µL)	Membrane	Intracellular
CD27	BV421	M-T271	BD	562513	2	All antibodies, except for CD154 BV605	
CD45RA	BV510	HI100	BD	563031	2.5		
CD154	BV605	24-31	BioLegend	310826	5		X
CD62L	BV650	DREG-56	BioLegend	304832	2.5		
CD127	BV711	HIL7RM21	BD	563165	2.5		
CD3	BV786	SK7	BD	563800	1		
CD25	FITC VioBright	4E3	Miltenyi	130-104-323	10		
CCR10	PerCP Cy5.5	1B5	BD	564772	2.5		
CXCR3	PE	1C6/CXCR3	BD	557185	10		
CCR6	PE-CF594	11A9	BD	564816	5		
CCR4	PE-Cy7	L291H4	BioLegend	359410	1		
CXCR5*	APC	51505	R&D	FAB190A-100	10		
CRCR5*	APC	REA103	Miltenyi	130-098-422	2.5		
CD45	AF700	HI30	BD	560566	2.5		
CD4	APC H7	SK3	BD	641398	5		

*Due to performance issues, we replaced of CXCR5 from R&D by CXCR5 from Miltenyi while the studies were ongoing.

Supplemental Table 4. Overview of all antibodies used in the EuroFlow PERISCOPE CD8 T-cell/NK cell panel. Official reference for this panel: Patent filed by Van Dongen et al. Means and Methods for Multiparameter Cytometry-Based Leukocyte Subsetting. P119646NL00 (2019). PCT/NL2020/050688, priority date 5 November 2019.

**Marker**	**Fluorochrome**	**Clone**	**Source**	**Cat. number**	**Volume (µL)**	**Membrane**	**Intracellular**
CD27	BV421	M-T271	BD	562513	2	X	
CD45RA	BV510	HI100	BD	563031	2.5	X	
CD154	BV605	24-31	BioLegend	310826	5		X
CD62L	BV650	DREG-56	BioLegend	304832	2.5	X	
CD16	BV711	3G8	BD	563127	2.5	X	
CD3	BV786	SK7	BD	563800	1	X	
CD57	FITC	HNK1	BD	333169	10	X	
CD28	PerCP Cy5.5	CD28.2	BioLegend	302922	5	X	
Granzyme B	PE	GB11	Sanquin	M2289	15		X
CD8	PE CF594	RPAT8	BD	562282	1	X	
TCRγδ	PE Cy7	11F2	BD	655410	1	X	
CD45	AF700	HI30	BD	560566	2.5	X	
CD56	APC H7	HCD56	BioLegend	318332	5	X	

Supplemental Table 5. Overview of all antibodies used in the EuroFlow PERISCOPE DC-Monocyte cell panel. Official references for this panel: Van der Pan et al, Development of a standardized and validated flow cytometry approach for monitoring of innate myeloid immune cells in human blood, Frontiers in Immunology, *2022:5141*. Patent filed by Van Dongen et al. Means and methods for multiparameter cytometry-based leukocyte subsetting. P119646NL00 (2019), PCT/NL2020/050688, priority date 5 November 2019.

Marker	Fluorochrome	Clone	Source	Cat. number	Volume (µL)	Membrane	Intracellular
CD141	BV421	1A4	BD	565321	2.5	X	No intracellular staining
CD62L	BV605	DREG-56	BioLegend	304833	5	X	
HLA DR	BV711	G46-6	BD	563696	2.5	X	
CD16	BV786	3G8	BD	563690	5	X	
CD1c	BB515	F10/21A3	BD	565054	5	X	
CD36	PerCP Cy5.5	CLB-IVC7	Immunostep	36PP5-100T	10	X	
SLAN	PE	DD.1	Miltenyi	130-093-029	10	X	
FcεR1	PE	AER-37	Thermo Fisher	A18416	5	X	
CD33	PE Cy7	P67.6	BD	333952	5	X	
CD300e (IREM2)	APC	UP-H2	Immunostep	IREM2A-100T	10	X	
CD303	APC	AC144	Miltenyi	130-090-905	10	X	
CD45	AF700	HI30	BD	560566	10	X	
CD14	APC H7	MϕP9	BD	641394	5	X	
Brilliant Stain Buffer			BD	566349	50	N/A	

Supplemental Table 6. Phenotypic descriptions used to define B-cell subsets stained with EuroFlow PERISCOPE B-cell and plasma cell panel (BIGH) panel by manual analysis. The removal of debris and doublets is not indicated in the analysis strategy below, but should be performed to ensure high quality data. This table was previously published in: Diks et al. Age and Primary Vaccination Background Influence the Plasma Cell Response to Pertussis Booster Vaccination. Vaccines, 2022.

Stepwise approach (gating in 2D plots)	Phenotypic description
#1. Identification of total plasma cells	CD45+CD19dimCD38highCD21-CD24-<br>Light scatter properties are low/medium (between lymphocytes and monocytes).
#2. Definition of maturation stage	• Least mature plasma cells: CD20+CD138-<br>• Intermediate mature plasma cells: CD20-CD138-<br>• Most mature plasma cells: CD20-CD138+
#3. Classification of plasma cells based on isotype	• IgM+, no expression of other isotype Igs<br>• IgG1+, no expression of other isotype Igs<br>• IgG2+, no expression of other isotype Igs<br>• IgG3+, no expression of other isotype Igs<br>• IgG4+, no expression of other isotype Igs<br>• IgA1+, no expression of other isotype Igs<br>• IgA2+, no expression of other isotype Igs<br>• IgD+, no expression of other isotype Igs<br>• IgH-, no Ig expression of any isotype Igs
#4. Classification of plasma cells based on CD62L expression	• CD62L-<br>• CD62L+
#5. Identification of total B cells	• CD45+CD19+CD20+ B cells show low light scatter characteristics (lymphocyte range)
#6. Identification of switched memory B-cell (MBC) subsets based on isotype. Switched MBCs express only one isotype	• IgG1+, no expression of other isotype Igs<br>• IgG2+, no expression of other isotype Igs<br>• IgG3+, no expression of other isotype Igs<br>• IgG4+, no expression of other isotype Igs<br>• IgA1+, no expression of other isotype Igs<br>• IgA2+, no expression of other isotype Igs
#7. Subclassification based on maturation/functional CD markers	• CD20+CD21+<br>○ Homogenous CD24 staining<br>• CD20++CD21-/dim<br>○ CD24+<br>○ CD24-
#8. Subclassification based on CD62L/CD27 positivity	• CD27+CD62L+<br>• CD27+CD62L-<br>• CD27-CD62L-<br>• CD27-CD62L+
#9. Identification of non-switched MBCs	CD27+IgM++IgD+<br>Of note, a minor subset of IgD+IgM- MBCs may be found as well. These can be classified separately.
#10. Subclassification based on maturation/functional CD markers	• CD20+CD21+<br>○ Homogenous CD24 staining<br>• CD20++CD21-/dim<br>○ CD24+<br>○ CD24-<br>No further subclassification in these populations.
#11. Classification of pre-germinal center (preGC) B cells	CD27-IgM+IgD+

#12. Subclassification based on maturation/functional CD markers	• Immature preGC B cells: CD38+CD24+CD5+CD21-/+<br>• Naive CD5+ B cells: CD38-/dim CD24-/dimCD5+<br>• Naive CD5- B cells:CD38-/CD24-/dimCD5-
#13. Subclassification of naive B cells based on maturation/functional CD markers	• CD20+CD21+<br>○ Homogenous CD24 staining<br>• CD20++CD21-/dim<br>○ CD24+<br>○ CD24-

Supplemental Table 7. Phenotypic descriptions used to define T-cell subsets stained with EuroFlow PERISCOPE CD4 T-cell (TCD4) panel by manual analysis. The removal of debris and doublets is not indicated in the analysis strategy below, but should be performed to ensure high quality data. Official reference for the used panel and these phenotypic descriptions: Botafogo V, Pérez-Andres M, Jara-Acevedo M, et al. Age distribution of multiple functionally relevant subsets of CD4+ T cells in human blood using a standardized and validated 14-color EuroFlow immune monitoring tube. *Frontiers in immunology.* 2020;11:166. And: Patent filed by Van Dongen et al. Means and Methods for Multiparameter Cytometry-Based Leukocyte Subsetting. P119646NL00 (2019). PCT/NL2020/050688, priority date 5 November 2019.

Stepwise approach (gating in 2D plots)	Phenotypic description
#1 Identification of total CD4 T cells	CD3+CD4+CD45+<br>Light scatter properties are low ('lymphocyte gate').
#2 Identification of regulatory T cells (Tregs) within CD4 T cells	CD25+CD127dim
#3 Identification of follicular helper T cells (TFHs) within CD4 T cells	CD25-/dim CD185+ CCR10-
#4 Division of total CD4 T cells into T-helper (TH) subsets	• Divide based on chemokine receptor expression (CD183, CD194, CD196, and CCR10)<br>o Naive: CD27+CD45RA+CD62L+CD127+CD183-CD194-CD196-CCR10-<br>o TH1: CD183+ CD194-CD196-CCR10-<br>o TH2: CD183- CD194+CD196-CCR10-<br>o TH17: CD183- CD194+CD196+CCR10-<br>o TH1/17: CD183+CD194-CD196+CCR10-<br>o TH22: CD183- CD194+CD196+CCR10+<br>o CD183+CD194+CD196+CCR10+<br>o CD183+CD194+CD196+CCR10-<br>o CD183+CD194+CD196-CCR10+<br>o CD183+CD194+CD196-CCR10-<br>o CD183+CD194-CD196+CCR10+<br>o CD183+CD194-CD196-CCR10+<br>o CD183-CD194-CD196+CCR10-<br>o CD183-CD194+CD196-CCR10+<br>o Non-naive CD4+CD183-CD194-CD196-CCR10-CD27-/+CD45RA-CD62L-/+
#5 Division of total Tregs in TH-like subsets	• Divide primarily based on chemokine receptor expression (CD183, CD194, CD196, and CCR10)<br>o Naive Treg: CD27+CD45RA+CD62L+ CD183-CD194-CD196-CCR10-<br>o TH1-like: CD183+CD194-CD196-CCR10-<br>o TH2-like: CD183-CD194+CD196-CCR10-<br>o TH17-like: CD183-CD194+CD196+CCR10-<br>o TH22-like: CD183-CD194+CD196+CCR10+<br>o CD183+CD194+CD196-CCR10+ Treg<br>o CD183+CD194+CD196-CCR10- Treg<br>o CD183+CD194+CD196+CCR10- Treg<br>o CD183+CD194+CD196+CCR10+ Treg<br>o CD183-CD194+CD196-CCR10+ Treg
#6 Division of total TFHs in Treg-/TH-like subsets	• Divide primarily based on chemokine receptor expression (CD183, CD194, CD196, and CCR10)<br>o CD185+CD27+CD45RA+CD62L+ T cells (CD183-CD194-CD196-CCR10-)<br>o Treg TFH: CD127+/dimCD183-/+CD194-/+CD196-/+CCR10-<br>o TH1-like: CD183+CD194-CD196-CCR10-

	○ TH2-like: CD183-CD194+CD196-CCR10-<br>○ TH17-like: CD183-CD194+CD196+CCR10-<br>○ TH1/17-like: CD183+CD194-CD196+CCR10-<br>○ CD183+CD194+CD196-CCR10- TFH<br>○ CD183+CD194+CD196+CCR10- TFH<br>○ CD183-CD194-CD196+CCR10- TFH<br>○ CD183+CD194+CD196-CCR10- TFH
#7 division of each subset into different maturation stage	• Divide based on CD27, CD45RA, and CD62L<br>○ Central memory (CD27+CD45RA-CD62L+)<br>○ Transitional memory (CD27+CD45RA-CD62L-)<br>○ Effector memory (CD27-CD45RA-CD62L-/+)<br>○ Terminal effector (CD27-CD45RA+CD62L-/+)

Supplemental Table 8. Phenotypic descriptions used to define T-cell and NK-cell subsets stained with the EuroFlow PERISCOPE CD8 cytotoxic T-cell (CYTOX) panel by manual analysis. The removal of debris and doublets is not indicated in the analysis strategy below but should be performed to ensure high quality data. Official reference for these phenotypic descriptions: Patent filed by Van Dongen et al. Means and Methods for Multiparameter Cytometry-Based Leukocyte Subsetting. P119646NL00 (2019). PCT/NL2020/050688, priority date 5 November 2019.

Stepwise approach (gating in 2D plots)	Phenotypic description
#1 Identification of total T cells	CD3+CD45+<br>Light scatter properties are low ('lymphocyte gate').
#2 Identification of total TCRγδ+ T cells within total T cells	CD3+ TCRγδ+
#3 Identification of total CD8+ and CD8- T cells within total T cells	CD8+ T cells: CD8+ TCRγδ-<br>CD8- T cells: CD8- TCRγδ-
#4 Identification of total NK cells	CD3- TCRγδ- CD45+<br>Light scatter properties are low ('lymphocyte gate').<br>CD56-/+<br>CD16-/+ (lower than neutrophils)<br>CD45RA-/+ (most NK cells are CD45RA positive)<br>CD62L-/+
# 5 Identification of additional lymphocytes	Light scatter properties are low ('lymphocyte gate').<br>CD45+CD3-CD4-
#6 Identification of myeloid cells	Neutrophils: high SSC, CD16+CD45+<br>Eosinophils: high SSC, CD45+, Autofluorescence results in double positive population in CD57 vs cy Granzyme B plot<br>Monocytes: intermediate SSC, CD45+CD16+/- CD45RA-/+ and mostly CD62L+
#7 Subsetting of TCRγδ+ T cells	Naive: CD27+CD28+CD45RA+CD62L+GranzB-CD57-<br>Central memory: CD27+CD28+CD45RA-CD62L+<br>- CD57-cyGranzB-/+<br>- CD57+CyGranzB+<br>Transitional memory: CD27+CD28-/+CD45RA-CD62L-/dim<br>- CD57-cyGranzB-<br>- CD57-CyGranzB+<br>- CD57+CyGranzB+<br>Peripheral memory: CD27-CD28+CD45RA-CD62L-/+<br>- CD57-cyGranzB-<br>- CD57-CyGranzB+<br>- CD57+CyGranzB+<br>Early effector: CD27+CD28-CD45RA+CD62L-/+<br>- CD57-CyGranzB+<br>- CD57+CyGranzB+<br>Terminal effector: CD27-CD28-CD45RA+CD62L-/+<br>- CD57-CyGranzB+<br>- CD57+CyGranzB+
#8 Subsetting of CD8+ T cells	Naive: CD27+CD28+CD45RA+CD62L+ (NB: in some donors the naive population can be divided into CD62Lhigh and CD62Llow)<br>Central memory: CD27+CD28+CD45RA-CD62L+<br>- CD57-CyGranzB-/+<br>- CD57+ CyGranzB-/+<br>Transitional memory:CD27+CD28+CD45RA-CD62L-/dim<br>- CD57-cyGranzB-<br>- CD57+CyGranzB-

	- CD57-CyGranzB+<br>- CD57+CyGranzB+<br>Peripheral memory: CD27-CD28-/+CD45RA-CD62L-/+<br>- CD57-cyGranzB-<br>- CD57-CyGranzB+<br>- CD57+CyGranzB+<br>Early effector: CD27+CD28-CD45RA+CD62L-/+<br>- CD57-cyGranzB-<br>- CD57-CyGranzB+<br>- CD57+CyGranzB+<br>Terminal effector: CD27-CD28-CD45RA+CD62L-/+<br>- CD57-cyGranzB-<br>- CD57-CyGranzB+<br>- CD57+CyGranzB+
#9 Subsetting of NK cells	CD56+ bright NK cells: CD56brightCD16lo/dim<br>- CD57-cyGranzB-<br>- CD57-CyGranzB+<br>CD56+dim NK cells: CD56dimCD16+<br>- CD57-cyGranzB-<br>- CD57-CyGranzB+<br>- CD57+CyGranzB+

Supplemental Table 9. Phenotypic descriptions used to define innate immune cell (sub)sets stained with the EuroFlow PERISCOPE DC-Monocyte panel by manual analysis. The removal of debris and doublets is not indicated in the analysis strategy below, but should be performed to ensure high quality data. Official references for this phenotypic description: Van der Pan et al, Development of a standardized and validated flow cytometry approach for monitoring of innate myeloid immune cells in human blood, Frontiers in Immunology, 2022, 5141. And: Patent filed by Van Dongen et al. Means and Methods for Multiparameter Cytometry-Based Leukocyte Subsetting. P119646NL00 (2019). PCT/NL2020/050688, priority date 5 November 2019.

Stepwise approach (gating in 2D plots)	Phenotypic description
#1 Identify eosinophils	SSC high, CD45+ neg. for all other markers in the panel
#2 Identify mature neutrophils	SSC high CD45+CD16+
#3 Identify immature neutrophils	CD45+CD33+CD16-/+HLA DR-CD14-SLAN&FcER1-<br>o CD62L-<br>o CD62L+
#4 Identify monocytes	SSC intermediate<br>CD45+CD33+CD16-/+HLA DR+CD14-/+SLAN&FcER1-/+
# 5 Divide the monocytes based on CD14/CD16 expression	ncMo: CD14-/dimCD16+CD62L- SLAN &FcER1-/+<br>o SLAN+CD36+<br>o SLAN-CD36+<br>o SLAN-CD36-<br>o SLAN+CD36-<br>iMo:CD16+CD14+HLA DR+Slan&FcER1-CD300e&CD303+CD36+<br>cMo:CD16-CD14+CD62L-/+<br>o CD62L+FcER1+<br>o CD62L-FcER1+<br>o CD62L+FcER1-<br>o CD62L-FcER1-
#6 Identify the CD1c+ myeloid DCs	SSC intermediate<br>CD45+CD33+CD141-/dimFcER1+HLA DR+CD16-CD14-/dim<br>- CD14dim<br>- CD14-
#7 Identify the plasmacytoid DCs	SSC intermediate<br>CD45+CD303+CD14-HLA DR+CD16-
#8 Identify the CD141+ myeloid DCs	CD141+CD33+CD300e-CD303-CD14-HLA DR+CD16-
#9 Identify the Axl+ DCs within the plasmacytoid DCs	CD33dimCD141+CD36dim
#10 Identify the basophils	SSC intermediate<br>CD45dimCD33+CD303-CD300e-CD14-HLA DR-CD62L+
# Identify 'unspecified nucleated cells'	Remaining CD45+ events that fit the singlet gate

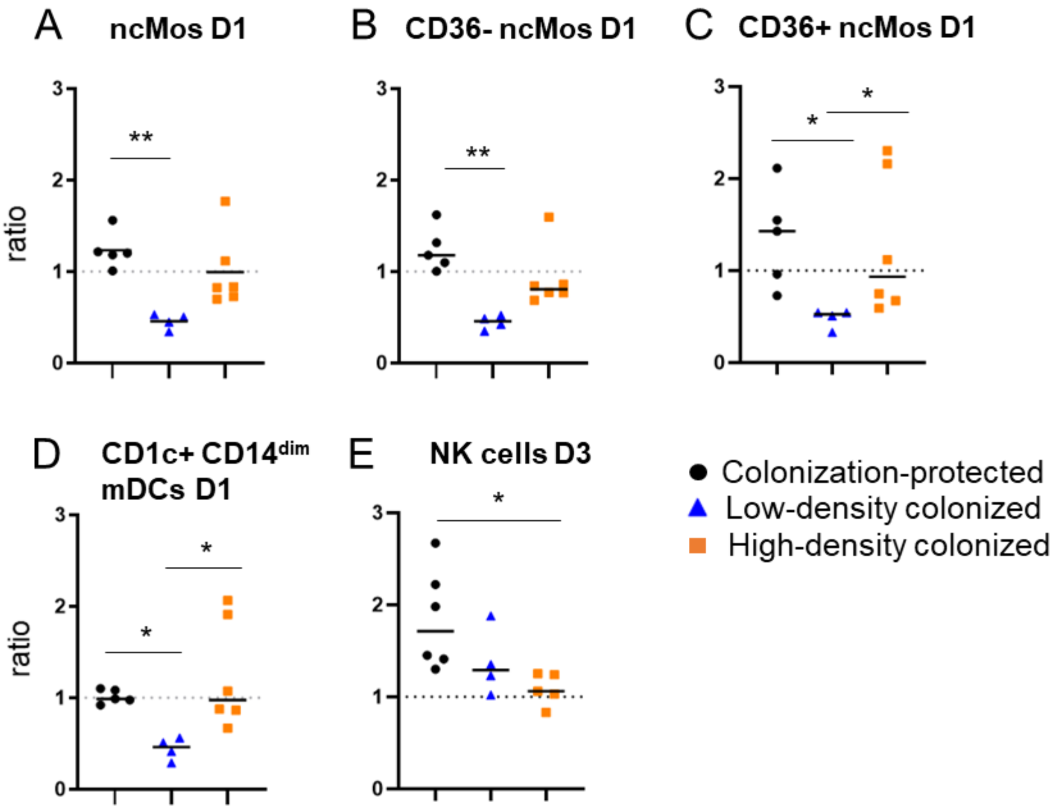

Supplemental Figure 1. Different kinetics in the innate immune cell compartment based on colonization density. Differences are presented as ratio of baseline. Dashed line indicates a ratio of 1.0 (baseline value). Kruskal-Wallis one way ANOVA followed by Dunns' test was used to assess differences. D= days after challenge. * $p<0.05$, ** $p<0.01$.

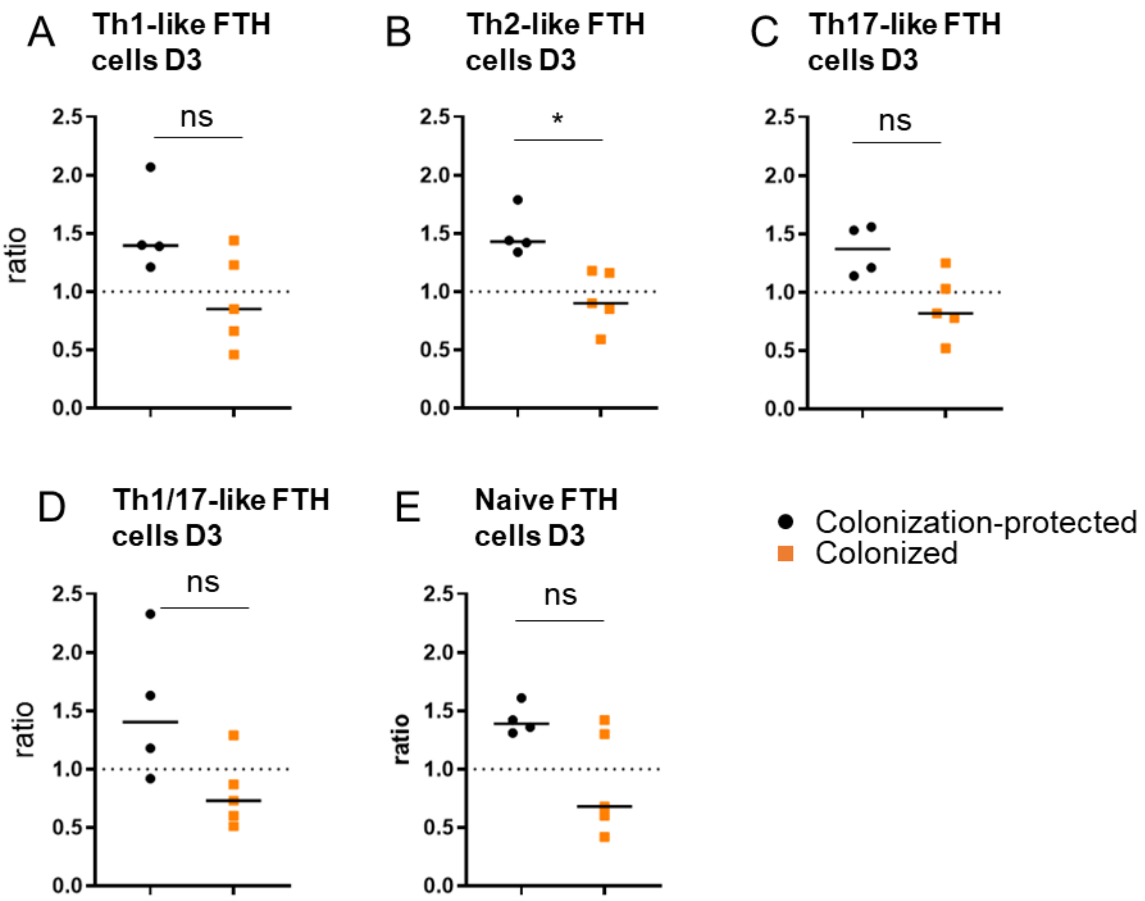

Supplemental Figure 2. Expansion of follicular T helper (FTH) cells in participants protected against colonization. Expansion of FTH subsets at d3 post-challenge expressed as ratio of baseline. Dashed line indicates a ratio of 1.0 (baseline value). Of note, due to technical limitations, in 6/15 participants no FTH subsets could be defined. N=9. Differences between groups were assessed using Mann-Whitney U test. * $p<0.05$. D= days after challenge.

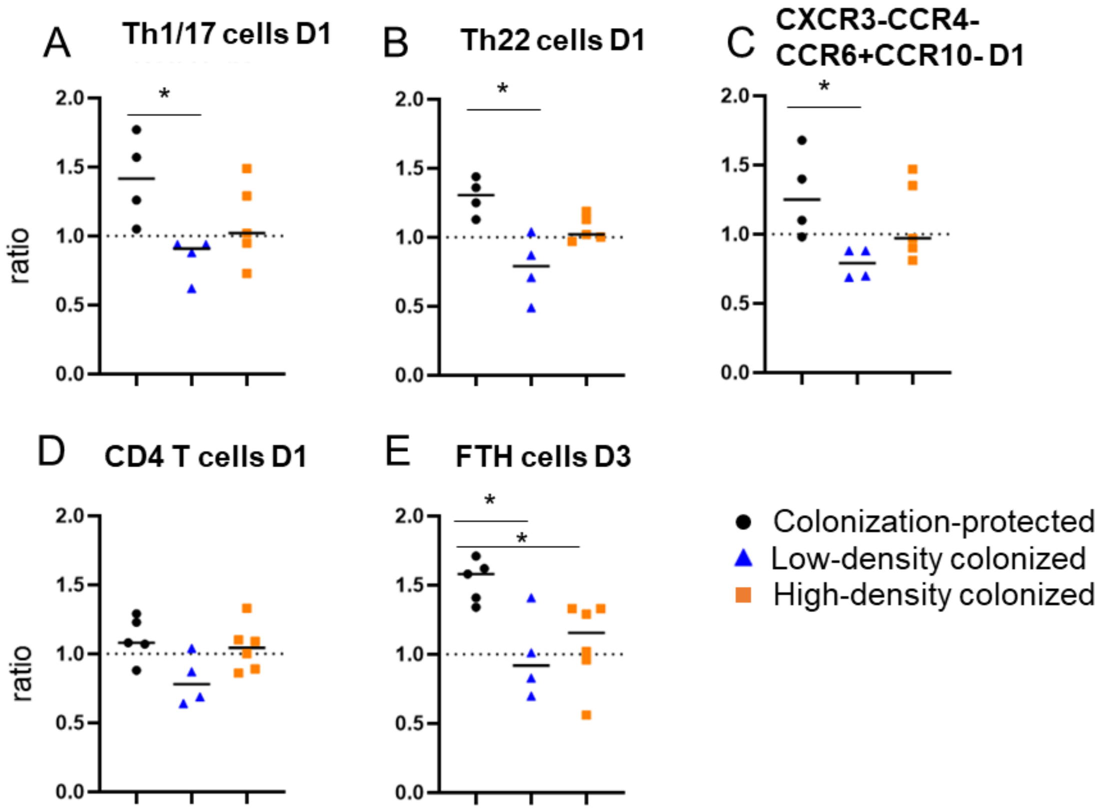

Supplemental Figure 3. Kinetics of T helper cells in participants grouped based on colonization density. Expansion of reduction of circulating cells was expressed as ratio of baseline. Dashed line indicates a ratio of 1.0 (baseline value). Kruskal-Wallis one way ANOVA followed by Dunns' test was used to assess differences. * $p<0.05$. D= days after challenge, FTH= follicular T helper cells, Th cell= T helper cell.

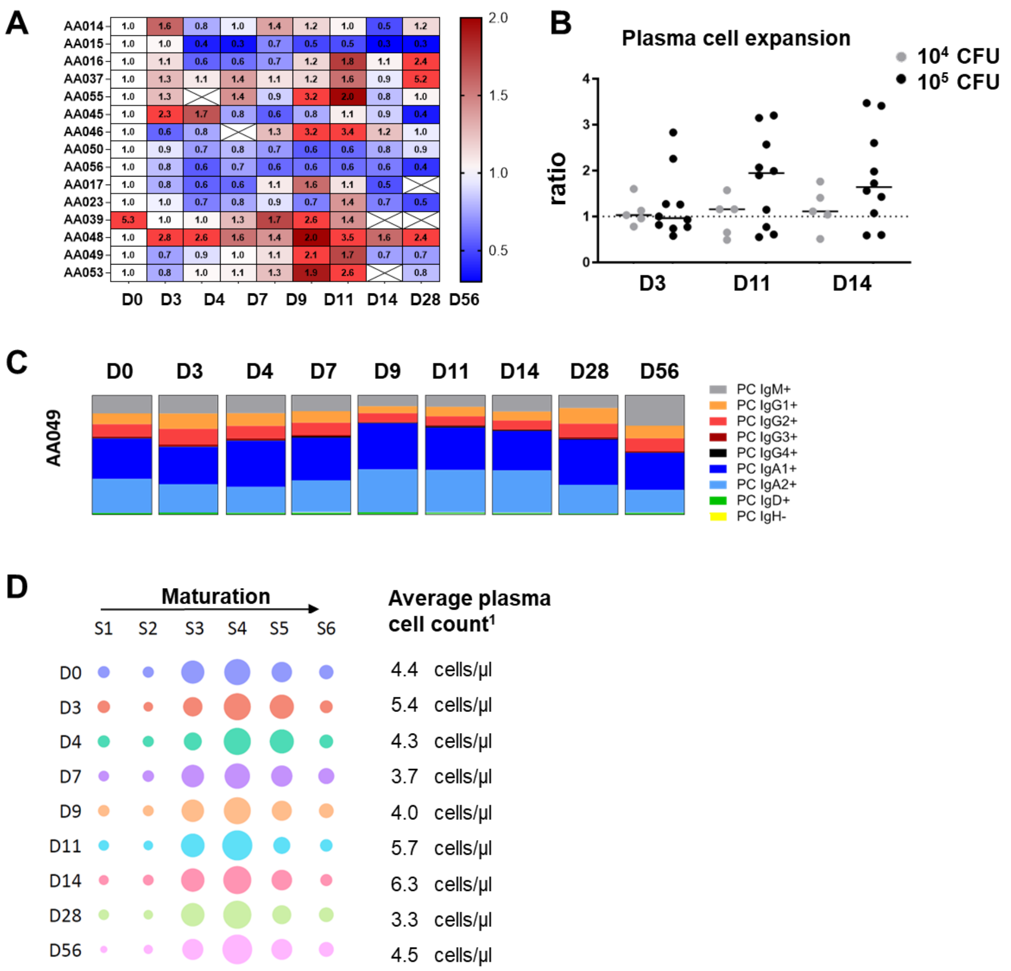

Supplemental Figure 4. Kinetics in the plasma cell compartment upon bacterial challenge. A. Heatmap representing the expansion of plasma cells in ratio compared to baseline ranked based on colonization status. NB: Participant ID.12 showed a strongly elevated number of total and IgM plasma cells at baseline. Therefore, it was decided to normalize the plasma cell numbers of this participant to d3 instead of baseline. **B.** Expansion of plasma cell in ratio compared to baseline. Dashed line indicates a ratio of 1.0 (baseline value).The cohort is split based on initial CFU dosage (10^4 or 10^5 CFU of BP1917) received at the day of challenge. Each dot represents one participant, the bar indicates the median value of all participants in each cohort. **C.** Fluctuations in the distribution of the plasma cell compartment over time. One representative participant is shown. **D.** Per time point the percentage of plasma cells in each maturation stage was plotted (total plasma cells in donors irrespective of their colonization status, grouped per time point). The size of the dot indicates the % of plasma cells in each maturation stage (average of participant). Cell count is shown at the right side of the plot (average of participants). Bubble plots were generated using plotly python graphing library.[1] Participant ID.12 was not included in panel D due to deviating baseline IgM plasma cell counts, possibly hinting at an ongoing immune response at time of challenge. D= Days after challenge.

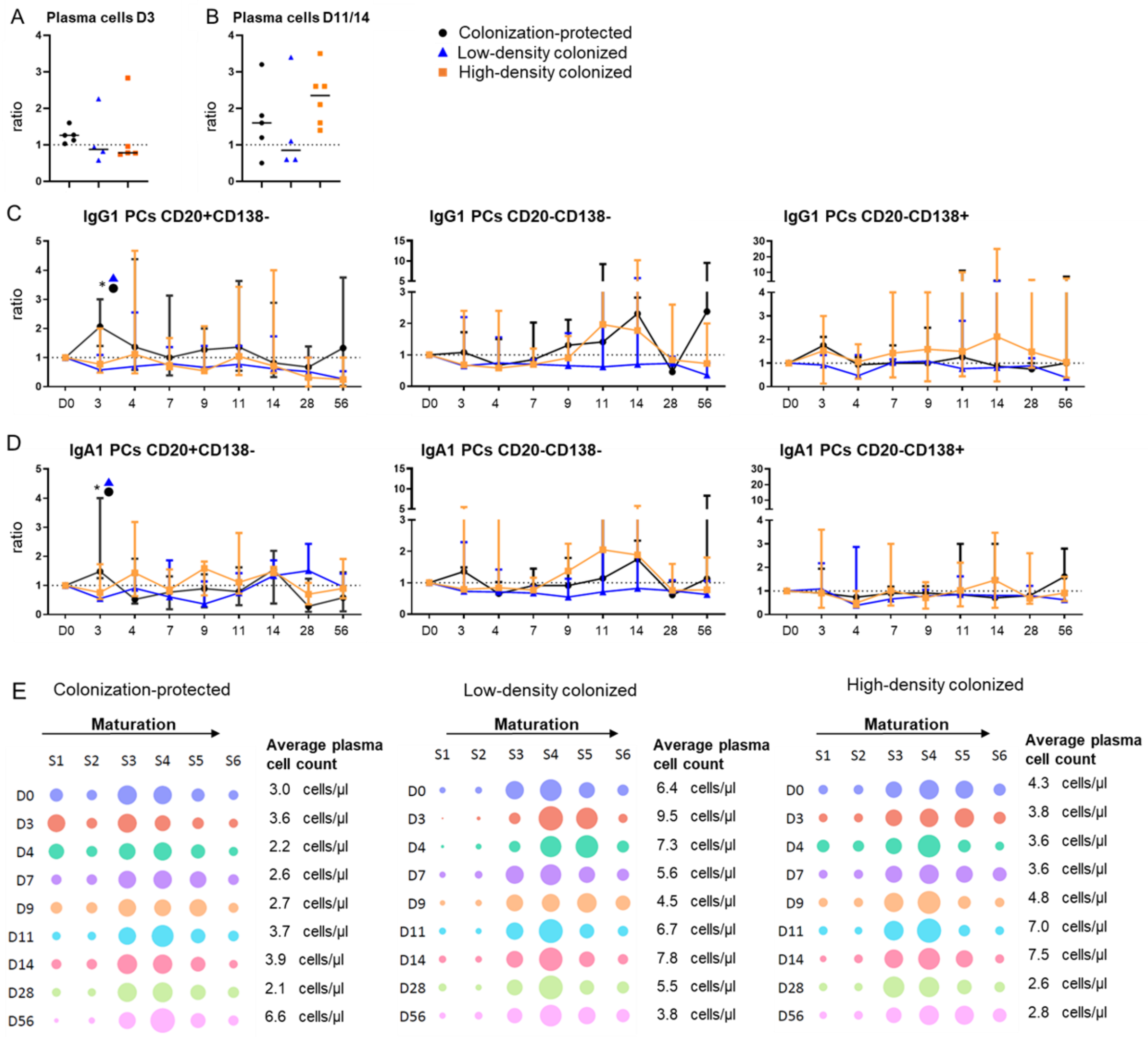

Supplemental Figure 5. Plasma cell kinetics in participants that were colonization-protected, low-density colonized, or high-density colonized. A. Plasma cell expansion at d3 post- challenge. Expansion expressed as ratio of baseline. Each dot represents one participant, the bar indicates the median value of all donors each cohort. **B.** Plasma cell expansion at d11/14 post-challenge. Expansion expressed as ratio of baseline. For panel A and B, Kruskal-Wallis one way ANOVA followed by Dunns' test was used to assess differences. **C+D.** Total plasma cells were divided into three different maturation stages (CD20+CD138-, CD20-CD138-, CD20-CD138+). Per maturation stage, the ratio of baseline was compared between donors of the three groups for IgG1 (**C**) and IgA1 (**D**) plasma cells. **E.** Per time point the percentage of plasma cells in each maturation stage was plotted (total plasma cells in donors that that were or were not colonized, grouped per time point). The size of the dot indicates the % of plasma cells in each maturation stage (average of donors). Cell count is shown at the right side of the plot (average of participants). Bubble plots were generated using plotly python graphing library. Dashed line indicates a ratio of 1.0 (baseline value). Statistical test performed for longitudinal analysis: Wilcoxon matched-pairs signed rank test. Corrected for multiple testing with Bonferroni correction. Statistical test for comparison between groups per time point; Mann-Whitney U test. D= days after challenge.

Supplemental Acknowledgement

Members of the PERISCOPE Consortium

Commissariat a l'energie atamique et aux energies alternatives: Contreras Vanessa, Le-Grand Roger, Mangeot Isabelle, Naninck Thibaut, Bosquet Nathalie, Chapon Catherine, Elies Frederic, Klimova Nela

Hospices Cantonaux CHUV: Fenwick Craig, Mercuri Davide, Pantaleo Giuseppe, Scherrer Christiane

European Research and Project Office: Haardt Jakob, Scheid Michaela, Schneider Klara, Wallrich Laura

GSK Biologicals SA: Cochard Landry, De Klerck Bert, Denoel Philippe, Deshpande Yashodhan, Dominici Debora, Feron Christiane, Mesaros Narcisa, Godfroid Fabrice, Leuzzi Rosanna, Ouaked Nadia, Pena-Gomez Carine, Stoffel Michel, van den Berg Robert, Van der Most Robbert, Pizza Mariagrazia, Siena Emilio, Silerova Marcela, Temmerman Stéphane, Tibaldi Fabian, Toffalini Federica, Vigano-Wolff Catherine, Walter Carsten

Imperial College of Science, Technology and Medicine: Kampmann Beate, Meads Carole, Rice Tom, Wheatley Scott

Mikrobiologicky USTAV-AVCR VVI: Dienstbier Ana, Holubova Jana, Kramlova Pavla, Linhartova Irena, Pěnek Tomáš, Sebo Peter, Vecerek Branislav, Krupickova Ilona, Schoffel Ondrej

Institut National de la Sante et de la recherce medicale: Coutte Loic

Institut Pasteur de Lille Fondation: Chatgnon Jonathan, Dubois Violaine, Jean Fabienne, Locht Camille, Mielcarek Nathalie, Raze Dominique, Berche Patrick, Lemoine Yves

London School of Hygiene and Tropical Medicine: Saso Anja

Leiden University Medical Center: Berkowska Magda, Bitter Marieke, Diks Annieck, Lubbers Bart, Teodosio Cristina, van Dongen Jacques J.M., Wieles Brigitte, Groenland Rick, De Mooij Bas

Medical Research Council (The Gambia): Johnson Njilan, Njie-Jobe Jainaba, Roetynck Sophie, Batchilly-Stanley Elisabeth

Department of Health, Public Health England: Cavell Breeze, Gorringe Andrew, Mauchline Margaret, Hanningan Bernie, Newton John, Rowe Cathy

Rijksintituut voor Volksgezondheid en Milieu: Berbers Guy, Bosch Thijs, Brummelman Jolanda, Buisman Annemarie, Feuerstein Oskar, Hartwigsen Marjon, Helm Kina, Jochemsen Petra, Lambert Nora, Linde Annieke, Versteegen Pauline, de Rond Lia, de Zeeuw-Brouwer Mary-lene, van Eijk Laura, van Els Cecile, van Gaans Jacqueline, van Gageldonk Pieter, van der Want Willemijn

Radboud University Medical Center: Baakman Coos, Diavatopoulos Dimitri, Eleveld Marc, Fröberg Janeri, Gillard Joshua, Huijnen Martijn, Sette Alessandro, Simonetti Elles, Venkatasubramanian Prashanna Balaji, de Groot Ronald, de Jonge Marien, van Leeuwen Evert, van Schuppen Evi, van den Brand Mariel, van Hughten Sandra, van Langen Maarten, van Hijum Sacha, Vriend Gert

Sanofi Pasteur SA: Ataman-Onal Yasemin, Blanc Pascal, Brown Monique, Carrington Krissy (Krishna), Chautard Emilie, Dauner Allison, Demont Clarisse, Jordanov Emilia, Liotard Marie, Macina Denis, Mistretta Noelle, Mosquera Luis, Rehm Christine, Reveneau Nathalie, Rokbi Bachra, Sarkar Sharmistha, Schanen Brian, Vargas Juan, Varghese Kucku, Amans Camille, Esteves Alex, Izquierdo Oleanda, Kester Kent, Lever David, ,Liu Yuanqing, Londono-Hayes Patricia, Ochs Martina, Oulerich Michel, Rokbi Bachra, Schaeffer Nathalie, Shaik Farzana, Wittman Vaughan

Trinity College Dublin: McPartlin Catherine, Murphy Heather, Borkner Lisa, Mills Kingston, Misiak Alicja, O'Reilly Lyn

University of Bath: Preston Andrew, Brian Kellie, Evans Alison

Universite libre de Bruxelles: Corbiere Veronique, Mascart Françoise, Leproult Rachel, Dirix Violette

Inversitaet Basel: Heininger Ulrich, Silfverberg Eve, Werka Elisabeth

University of Oxford: Bates Louise, Bibi Sagida, Clutterbuck Elizabeth, Demissie Tesfaye, Kelly Dominic, Koleva Stanislava, Negrete Ivette, Owino Nelly, Pollard Andrew, Robinson Hannah, Valente Pinto Marta, White Rachel, Boyer Charlotte, Holmes Alex, Wells Gill

University of Southampton: Mills Emma, Hill Alison, Ibrahim Muktar, Read Robert, de Graaf Hans

University of Salamanca: Corchado Juan Manuel, Gil Maria Jose, Almeida Julia, Mateos Alfredo, Orfao Alberto, Pérez-Andrés Martin, Ruiz Zapatero Esther, Damasceno Daniela, Hernandez-Delgado Alejandro, Botafogo Vitor

University of Turku: Barkoff Alex-Mikael, He Qiushui, Knuutila Aapo, Laakso Elisa, Mertsola Jussi, Teräsjärvi Johanna, Ewart Liisa, Suominen Kalle-Antti, Virta Mari

Q-Biologicals NV: Clauwaert Karine, van Broekhoven Annie, Vandenbussche Carolien
